# Chloroplast phylogenomic analyses reveal the deepest-branching lineage of the Chlorophyta, Palmophyllophyceae class. nov.

**DOI:** 10.1038/srep25367

**Published:** 2016-05-09

**Authors:** Frederik Leliaert, Ana Tronholm, Claude Lemieux, Monique Turmel, Michael S. DePriest, Debashish Bhattacharya, Kenneth G. Karol, Suzanne Fredericq, Frederick W. Zechman, Juan M. Lopez-Bautista

**Affiliations:** 1 Department of Biological Sciences, The University of Alabama, Tuscaloosa, AL 35484-0345, USA; 2 Department of Biology, Ghent University, 9000 Ghent, Belgium; 3 Southeast Environmental Research Center, Florida International University, Miami, FL 33199, USA; 4 Institut de biologie intégrative et des systèmes, Département de biochimie, de microbiologie et de bio-informatique, Université Laval, Québec (QC) Canada; 5 Department of Ecology, Evolution and Natural Resources and Department of Marine and Coastal Sciences, Rutgers University, New Brunswick, NJ 08901, USA; 6 Lewis B. and Dorothy Cullman Program for Molecular Systematic Studies, The New York Botanical Garden, Bronx, New York 10458, USA; 7 Department of Biology, University of Louisiana at Lafayette, LA 70504-3602, USA; 8 College of Natural Resources and Sciences, Humboldt State University, Arcata, CA 95521, USA

## Abstract

The green plants (Viridiplantae) are an ancient group of eukaryotes comprising two main clades: the Chlorophyta, which includes a wide diversity of green algae, and the Streptophyta, which consists of freshwater green algae and the land plants. The early-diverging lineages of the Viridiplantae comprise unicellular algae, and multicellularity has evolved independently in the two clades. Recent molecular data have revealed an unrecognized early-diverging lineage of green plants, the Palmophyllales, with a unique form of multicellularity, and typically found in deep water. The phylogenetic position of this enigmatic group, however, remained uncertain. Here we elucidate the evolutionary affinity of the Palmophyllales using chloroplast genomic, and nuclear rDNA data. Phylogenetic analyses firmly place the palmophyllalean *Verdigellas peltata* along with species of Prasinococcales (prasinophyte clade VI) in the deepest-branching clade of the Chlorophyta. The small, compact and intronless chloroplast genome (cpDNA) of *V. peltata* shows striking similarities in gene content and organization with the cpDNAs of Prasinococcales and the streptophyte *Mesostigma viride*, indicating that cpDNA architecture has been extremely well conserved in these deep-branching lineages of green plants. The phylogenetic distinctness of the Palmophyllales-Prasinococcales clade, characterized by unique ultrastructural features, warrants recognition of a new class of green plants, Palmophyllophyceae class. nov.

The green plants or Viridiplantae are an ancient and diverse group of photosynthetic eukaryotes. Molecular phylogenetic and ultrastructural data indicate that the green plants split early in their evolution (estimated between 800 and 1200 Mya) into two main clades: the Chlorophyta and Streptophyta^1^.

The Streptophyta include a diverse array of unicellular and multicellular green algae from freshwater environments (collectively termed the charophytes), and the land plants^2^,^3^. Phylogenomic analyses indicate that morphologically simple species of the Mesostigmatophyceae (*Mesostigma viride*, biflagellate unicells), Chlorokybophyceae (*Chlorokybus atmophyticus*, packets of non-motile cells), and Klebsormidiophyceae (packets of cells or simple filaments), characterized by cell division by furrowing, form the earliest-diverging clades of the Streptophyta^4^,^5^. The later-diverging clades of the Streptophyta evolved a new mechanism of cell division that involved the production of a phragmoplast, and cell-walls with plasmodesmata, which facilitated communication between cells, and ultimately development of complex tissues and plant bodies^6^.

The Chlorophyta include a large diversity of marine, freshwater and terrestrial green algae with a wide variety of morphologies, ranging from unicellular to complex multicellular morphologies. A paraphyletic assemblage of planktonic unicellular, mainly marine green algae forms the early-diverging clades of Chlorophyta, collectively called the prasinophytes. The early-diverging nature of these clades is reflected in a wide diversity of cellular architectures (including flagellate and coccoid cells that are naked, or covered by cell walls or organic body scales), flagellar behaviour, mitotic and cytokinetic processes, biochemical features, and photosynthetic pigments^7^,^8^,^9^,^10^,^11^,^12^,^13^. About ten prasinophyte clades have been identified based on nuclear-encoded small subunit (18S) rDNA sequences^9^,^10^,^11^,13–15. The affinities among these clades, however, were poorly resolved in 18S rDNA phylogenies, and only recently have phylogenetic relationships been elucidated with more confidence using multi-gene datasets^16^. The prasinophytes gave rise to the core Chlorophyta, which include unicellular and multicellular species that abound in marine, freshwater and terrestrial habitats. This clade includes the three species-rich classes Ulvophyceae, Trebouxiophyceae and Chlorophyceae, and two smaller classes Pedinophyceae and Chlorodendrophyceae^1^,^17^,^18^.

It is generally accepted that that the ancestral green plants were unicellular green algae with characters typical of most extant prasinophytes, such as the presence of flagella and organic body scales^1^,^19^. The nature of this hypothetical ancestral green flagellate, however, has been a matter of debate^9^,^19^. A better understanding of the diversity and phylogenetic relationships among early-diverging clades of Chlorophyta and Streptophyta is thus central to understanding the evolution of green plants.

The viewpoint that the earliest-diverging green plant lineages comprise green algae with simple morphologies was recently challenged by a molecular phylogenetic study by Zechman *et al.*^20^, which identified a deep-branching clade of macroscopic algae, the Palmophyllales. The clade includes the genera *Palmophyllum*, *Verdigellas* and *Palmoclathrus*, which occur in marine deep water and other dimly lit environments (*Verdigellas* has been recorded from depths down to 200 m)^20^. Species of Palmophyllales exhibit a unique type of multicellularity, forming macroscopic plants that are composed of isolated and undifferentiated spherical cells embedded in an apparently amorphous gelatinous matrix^21^,^22^,^23^,^24^. Although the Palmophyllales were identified as a distinct clade of green algae, the exact phylogenetic placement could not be determined with certainty. Analysis of the plastid genes *atpB* and *rbcL* placed the Palmophyllales sister to the Chlorophyta. On the other hand, analysis of nuclear 18S rDNA sequences allied the Palmophyllales with the Prasinococcales (a group of coccoid prasinophytes) in a clade of uncertain position.

The multiple genes encoded in the chloroplast genome (cpDNA) represent an invaluable source of data for resolving difficult phylogenetic questions, including deep relationships in green plants^16^,^17^,^25^,^26^,^27^. In addition, comparative analysis of chloroplast genomes from early-diverging green plants (prasinophytes and early-diverging streptophytes) provides important insights into the ancestral architecture and evolution of plastid genomes in the green plants^15^,^16^,^28^.

The aim of this study was to resolve the evolutionary affinities of the enigmatic Palmophyllales using a phylogenomic approach. We obtained the complete nucleotide sequence of the chloroplast genome of *Verdigellas peltata*, and performed multi-gene phylogenetic and comparative genomic analyses. In addition, we inferred phylogenies based on nuclear-encoded small and large subunit rDNA sequences, providing independent phylogenetic evidence. Our phylogenomic analyses firmly placed the Palmophyllales together with the Prasinococcales in the earliest-diverging lineage of the Chlorophyta. Comparative chloroplast genomic analyses provided new insights into the ancestral plastid genome of the green plants.

## Results and Discussion

### The cpDNA of *Verdigellas peltata* is small and highly compacted

**Figure 1:**
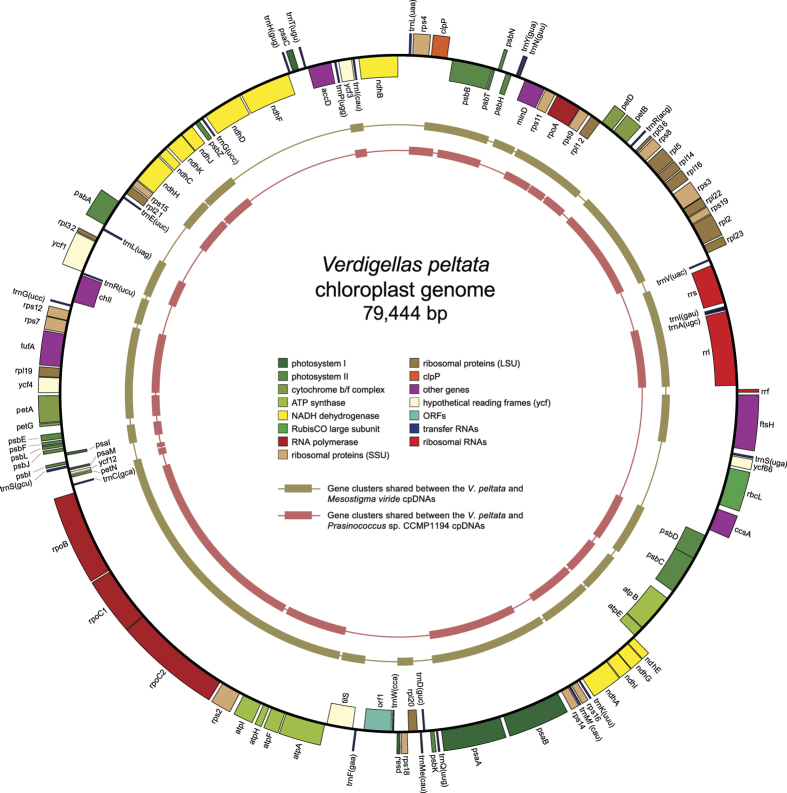
Gene map of the chloroplast genome of *Verdigellas peltata*. Genes shown on the outside of the circle are transcribed counterclockwise. Genes are coloured according to the functional categories shown in the legend inside the gene map. Thick lines in the inner rings represent conserved gene clusters between the cpDNAs of *V. peltata* and *Mesostigma viride*^28^, and between *V. peltata* and *Prasinococcus* sp. CCMP 1194^16^.

The circular chloroplast genome of *Verdigellas peltata* (Fig. 1) is 79,444 bp long, which is smaller than most chloroplast DNAs (cpDNAs) of free-living green algae^1^,^29^, but in the range of published prasinophyte cpDNAs^15^,^16^. GC content is 27.7%, which is the lowest value observed among the early-diverging chlorophytes examined so far. The cpDNA of the clade VI prasinophyte *Prasinococcus* sp. CCMP 1194 displays the second lowest value (32.1%)^16^. Similar to the situation in most prasinophytes and several other green algae, the *V. peltata* cpDNA lacks a large inverted repeat encoding the rRNA operon.

The genes of the *V. peltata* cpDNA are densely packed, with intergenic spacers accounting for only 13% of the total genome. Introns are absent, similar to the situation in the cpDNAs of the clade VI prasinophytes, Prasinophyceae sp. CCMP 1205^16^, *Nephroselmis olivacea*^30^, and *Micromonas* sp. RCC 299^31^. Chloroplast genomes of similar compactness have been found in small-celled prasinophytes, and this has been attributed to a strong selection pressure to maintain a small and compact chloroplast genome in picoplanktonic species^15^,^16^,^32^. The presence of a small and gene-dense cpDNA in *Verdigellas*, and the observation of compact cpDNAs in marine green macro-algae of the class Ulvophyceae^33^,^34^ indicate that highly compacted cpDNAs are not restricted to picoplanktonic species.

We identified 113 unique genes, including 85 protein-coding genes, 25 tRNA genes (*trnG*(ucc) is duplicated), and three rRNA genes. In addition, one freestanding open reading frame (ORF) of 1032 bp (*orf1*) was identified that did not show any relationship with known plastid genes. A blastp search indicated that this ORF contains a DNA polymerase III-like domain of bacterial origin (E-value 4e-25). The presence of bacterial genes in plastid genomes, possibly acquired through horizontal gene transfer, has only been observed in a few algal species, including the prasinophyte *Nephroselmis olivacea*^15^,^30^,^33^,^35^. It is relevant to note that *Verdigellas* harbours endophytic cyanobacteria (and probably a diverse community of other bacteria) in the gelatinous matrix of the thallus^24^. This close association may facilitate gene transfer from the endophytic bacteria to the host genome.

### The *V. peltata* cpDNA shows high similarities in genome organization and gene content with the cpDNAs of Prasinococcales and early-diverging Streptophyta

**Figure 2:**
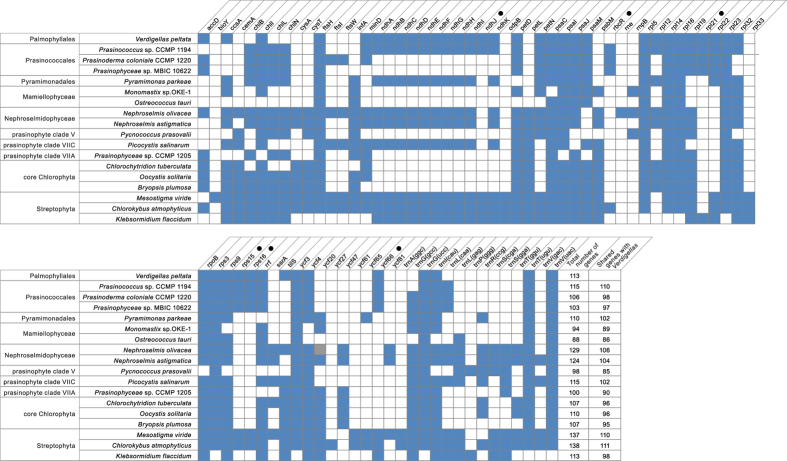
Comparison of gene contents between the cpDNA of *Verdigellas peltata* and a representative selection of published cpDNAs from prasinophytes, core Chlorophyta and early-diverging Streptophyta. The black circles denote the genes shared exclusively between the Streptophyta and at least one species of Palmophyllophyceae (Palmophyllales-Prasinococcales). The grey square indicates a pseudogene. The 68 genes present in all compared cpDNAs are not shown in the figure: *atpA*, *B*, *E*, *F*, *H*, *I*, *clpP*, *petA*, *B*, *G*, *psaA*, *B*, *psbA*, *B*, *C*, *D*, *E*, *F*, *H*, *I*, *J*, *K*, *L*, *N*, *T*, *Z*, *rbcL*, *rpl2*, *20*, *36*, *rpoA*, *C1*, *C2*, *rps2*, *4*, *7*, *8*, *11*, *12*, *14*, *18*, *19*, *rrl*, *rrs*, *tufA*, *ycf1*, *12*, and 21 tRNA genes: *trnA*(ugc), *C*(gca), *D*(guc), *E*(uuc), *F*(gaa), *H(*gug), *I(*gau), *K(*uuu), *L(*uag), *L(uaa)*, *Me*(cau), *Mf*(cau), *N*(guu), *P*(ugg), *Q*(uug), *R*(acg), *R*(ucu), *S*(gcu), *S*(uga), *W*(cca) and *Y*(gua). Data sources:^4^,15,^16^,^25^,^28^,^30^,^32^,^33^,^36^,^68^.

A comparison of gene repertoires between *V. peltata* and a representative selection of published cpDNAs from prasinophytes, core Chlorophyta and early-diverging Streptophyta is shown in Fig. 2. A total of 68 genes are shared among these 18 cpDNAs (see legend Fig. 2). *Verdigellas* shares the largest number of genes with the early-diverging streptophytes *Chlorokybus atmophyticus* (111 shared genes) and *Mesostigma viride* (110 shared genes) and the prasinophyte *Prasinococcus* sp. CCMP 1194 (110 shared genes). *Verdigellas* and several species of Prasinococcales share a unique set of five genes that is not found in other Chlorophyta cpDNAs: *ndhJ, rpl21, rps15, rps16* and *ycf66*. This set of chloroplast genes that was previously only known from the Prasinococcales and some Streptophyta was seen as support that these lineages maintain some ancestral genomic features of green algae^16^. Besides *ndhJ*, the *Verdigellas* cpDNA contains genes coding for 10 other subunits homologous to the mitochondrial NADH:ubiquinone oxidoreductase. In the Chlorophyta, the latter set of *ndh* genes has until now only been found in *Prasinococcus* sp. CCMP 1194, *Pyramimonas parkeae*, two *Nephroselmis* species, and *Picocystis salinarum*^16^. Common green algal chloroplast genes that are apparently absent from the *V. peltata* and prasinophyte clade VI cpDNAs include *psbM*, *infA* and *petL*.

**Figure 3:**
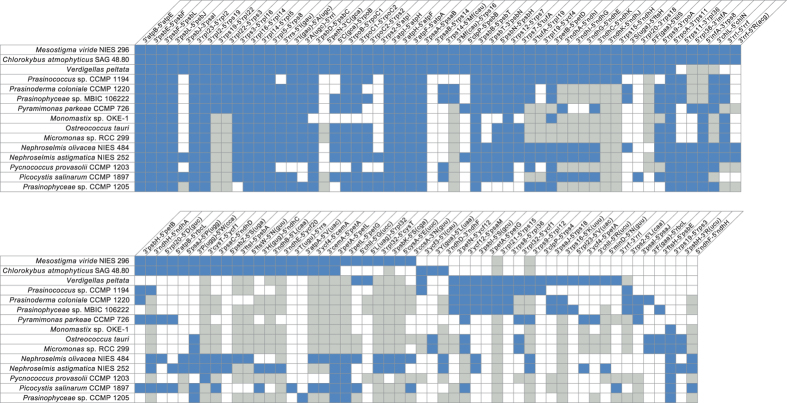
Shared gene pairs in the chloroplast genomes of early-diverging green algae. The gene pairs shared by at least three taxa were identified among all possible signed gene pairs in the compared genomes. Note that the *Verdigellas* gene pairs shared with only one taxon were not excluded. The presence of a gene pair is denoted by a blue box; a grey box refers to a gene pair in which at least one gene is missing due to gene loss.

As highlighted by our analyses of chloroplast gene pairs shared between *Verdigellas* and early-diverging green plants, retention of ancestral gene order appears to be the most interesting feature of the *Verdigellas* genome (Fig. 3). Indeed, among the prasinophytes examined thus far, *Verdigellas* shares the most gene pairs with the streptophytes *Mesostigma viride* and *Chlorokybus atmophyticus*. It even exhibits a higher level of synteny with *Mesostigma* than with any other clade VI prasinophyte taxa. A total of 81 *Verdigellas* genes form 20 clusters with *Mesostigma* (Fig. 1), whereas only 59–62 genes present in 16 clusters are conserved in the three other clade VI taxa. Of the latter taxa, *Prasinococcus* sp. CCMP 1194 displays the most similar gene order to the *Verdigellas* genome, with 22 syntenic blocks involving 69 genes (Fig. 1); however, this conservation level is not much different from those observed in the comparisons with Prasinophyceae sp. MBIC10622 (21 blocks, 68 genes) and *Prasinoderma coloniale* (19 blocks, 62 genes).

The *Verdigellas*/*Mesostigma* gene clusters clearly encompass a larger portion of the *Verdigellas* genome than the *Verdigellas*/*Prasinococcus* clusters (Fig. 1). The clusters in these two pairs of genomes have 23 endpoints in common; 15 of the 21 remaining *Verdigellas*/*Prasinococcus* endpoints interrupt *Verdigellas*/*Mesostigma* clusters, whereas only two of the 17 unique *Verdigellas*/*Mesostigma* endpoints interrupt *Verdigellas*/*Prasinococcus* clusters. These observations provide further evidence that ancestral gene order was disrupted more extensively in the Prasinococcales than in the Palmophyllales.

Like *Prasinococcus*, *Verdigellas* has not maintained an intact rDNA operon, but the two algal species do not share the same breakage site in this operon (Fig. 3, between *rrl* and *rrf* in *Verdigellas* and between *rrs* and *trnI*(gau) in *Prasinococcus*). While a number of IR-less green algal genomes have also been found to have a disrupted rDNA operon^15^,^16^,^36^, there are several cases of IR-less genomes that have preserved an intact operon (e.g. the prasinophyte *Monomastix* sp. OKE-1).

### The Palmophyllales-Prasinococcales clade forms the deepest branch of the Chlorophyta

**Figure 4:**
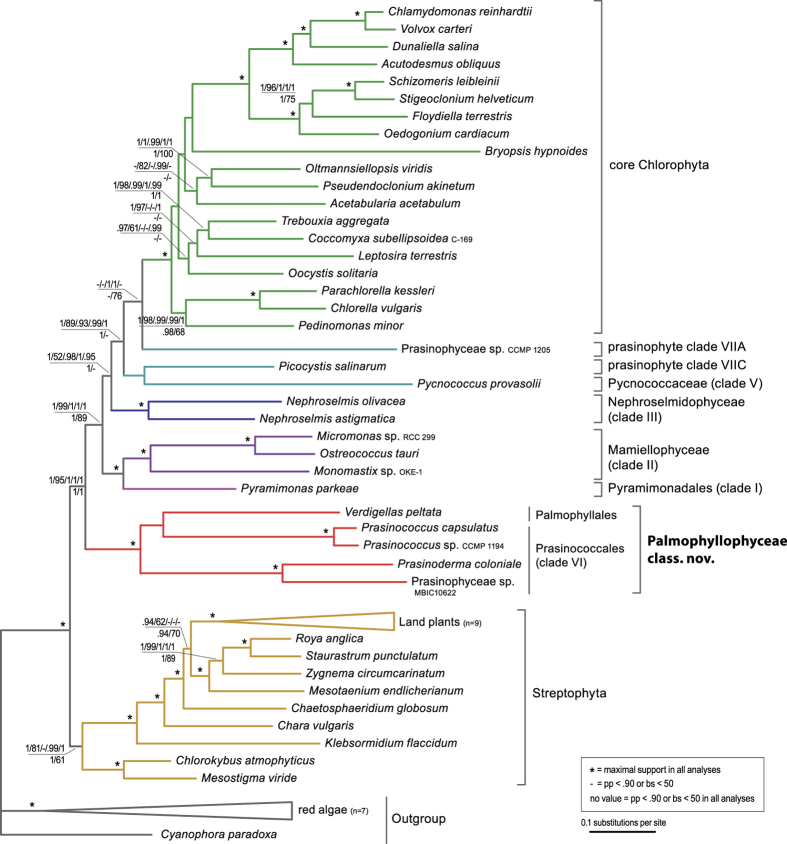
Plastid tree of green plants showing the phylogenetic position of the new class Palmophyllophyceae. Bayesian and ML phylogenies were inferred from 71 concatenated plastid genes and their translation products. The Bayesian majority-rule consensus tree resulting from the analysis of the AA alignment (13,730 amino acid positions) under the cpREV + Γ4 + F model is represented. Bayesian pp and ML bs values are shown above the branches for the analyses of the AA alignment; from left to right are indicated the pp and bs values for the analyses under the cpREV + Γ4 + F model, and the pp values for the PhyloBayes analyses under the CAT + Γ4 and CATGTR + Γ4 models, and the analysis of the Dayhoff6 recoded AA dataset using a homogeneous GTR + Γ4 model. Bayesian pp and ML bs values are shown below the branches for the nucleotide analyses (1st and 2nd codon position: 29,662 positions) under the GTR + Γ4 + I model with a partitioning strategy in which codon positions were treated separately (2 partitions). Asterisks indicate full support in all analyses; dashes denote pp values <0.90 or bs values <50. All inferred plastid trees are shown in the Supplementary Figs S2–S8.

Phylogenies were inferred from 71 concatenated plastid genes and their translation products. The Bayesian phylogeny inferred from amino acid (AA) sequences under the cpREV + Γ4 + F model is shown in Fig. 4 with indication of Bayesian posterior probability (pp) and maximum likelihood (ML) bootstrap support (bs) values, branch support from the analysis using the site-heterogeneous CAT + Γ4 and CATGTR + Γ4 models of evolution, and the analysis of the Dayhoff6 recoded AA dataset using a homogeneous GTR + Γ4 model, and branch support from the analyses of the nucleotide sequences (first two codon-positions). All inferred trees are shown in the Supplementary Figs S2–8. Overall, the topologies of the AA and nucleotide trees were congruent. The topology of the tree shown in Fig. 4, and in particular the branching order of the prasinophyte clades is in general agreement with published plastid phylogenies of green algae^16^,^17^. In all plastid gene analyses, *Verdigellas peltata* (Palmophyllales) forms a fully supported clade with four species of Prasinococcales (prasinophyte clade VI): *Prasinococcus capsulatus*, *Prasinococcus* sp. CCMP 1194, *Prasinoderma coloniale*, and Prasinophyceae sp. MBIC10622. The Palmophyllales-Prasinococcales clade was recovered as the sister group to all other Chlorophyta with high support in all analyses. These results are similar to chloroplast and 18S rDNA phylogenies^11^,^16^,^17^, which showed the early-diverging position of the Prasinococcales in the Chlorophyta. Within the Palmophyllales-Prasinococcales clade, the alliance of *V. peltata* with the Prasinococcales species received no support in the AA trees, while in the nucleotide trees *V. peltata* is sister to the four other species (pp = 0.95, bs = 94). Our phylogenomic results are thus in contrast with the plastid gene phylogeny of Zechman *et al.*^20^, who recovered the Palmophyllales as a sister clade to all other Chlorophyta with moderate support (pp = 0.97, bs = 77). This difference in topology is likely related to scarce phylogenetic information in two plastid genes (*rbcL* and *atpB*), and the missing *atpB* data for most prasinophytes in Zechman *et al.*^20^.

**Figure 5:**
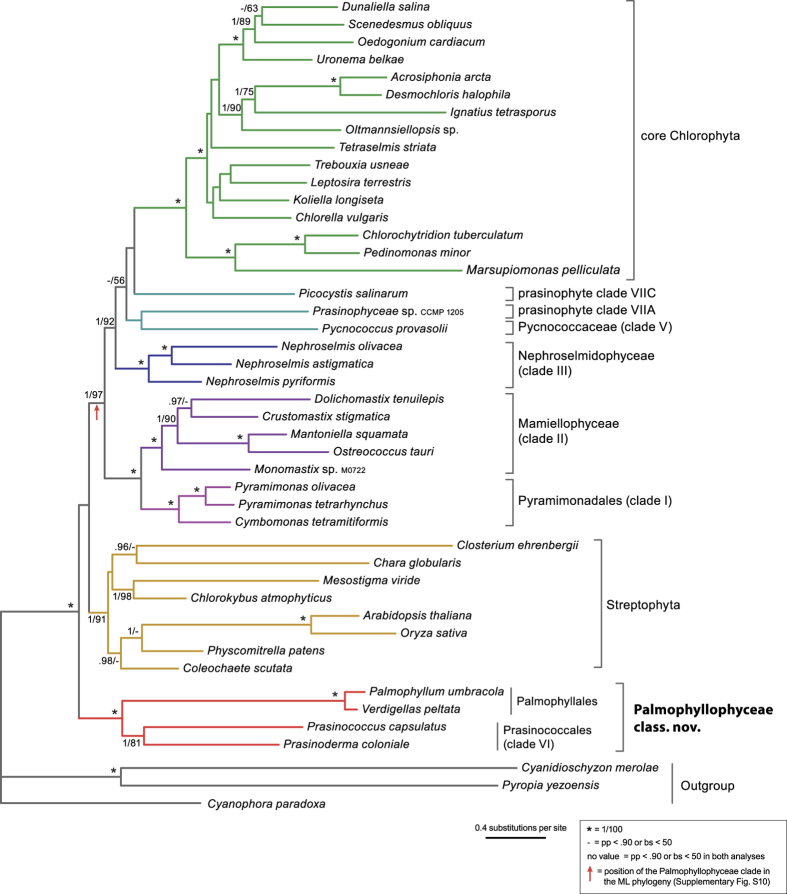
Nuclear rDNA tree of green plants showing the phylogenetic position of the new class Palmophyllophyceae. Bayesian and ML phylogenies were inferred from concatenated small (18S) and large (28S) subunit rRNA genes (4,579 nucleotide positions) under the GTR + Γ4 + I model with a partitioning strategy in which the 18S and 28S rDNA were treated separately. The Bayesian majority-rule consensus tree is represented. Bayesian pp and ML bs values are shown at the nodes. Asterisks indicate full support in both analyses; dashes denote pp values <0.90 or bs values <50. The red arrow indicates the position of the Palmophyllophyceae clade in the ML phylogeny (Supplementary Fig. S10).

The phylogenetic trees resulting from the analyses of the nuclear rDNA data (concatenated small and large subunit rRNA gene sequences) are summarized in Fig. 5. In general, the phylogenetic relationships are congruent with the plastid trees, although relationships among several prasinophyte clades received less support. As observed in the plastid tree, the Palmophyllales (*Verdigellas peltata* and *Palmophyllum umbracola*) form a fully supported clade with species of Prasinococcales (*Prasinococcus capsulatus* and *Prasinoderma coloniale*). Within this clade, the Palmophyllales and Prasinococcales represent two distinct subclades. Unlike the plastid phylogeny, the position of the Palmophyllales-Prasinococcales clade could not be determined with certainty. In the Bayesian tree, this clade is sister to the Chlorophyta-Streptophyta, while in the ML tree the Palmophyllales-Prasinococcales clade forms the earliest-diverging clade of the Chlorophyta, as in the plastid trees (red arrow in Fig. 5); however, both relationships received no statistical support (Supplementary Figs S9 and S10). Thus, the phylogenetic position of the clade has to be interpreted as unresolved based on the nuclear rDNA data, similar to the 18S phylogeny of Zechman *et al.*^20^. It should be noted that in some published 18S-based phylogenies with larger taxon sampling (but without members of Palmophyllales), the Prasinococcales have been resolved as an early-diverging clade of the Chlorophyta with low to moderate support^11^,^14^,^15^.

**Figure 6:**
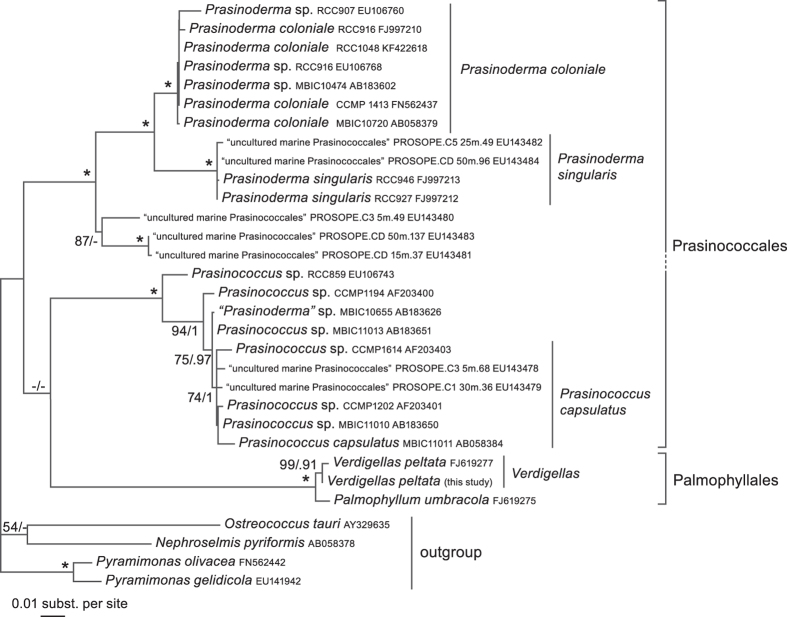
Phylogenetic tree illustrating the diversity within the Palmophyllophyceae based on nuclear 18S rDNA sequences. The best ML tree recovered under the GTR + Γ4 + I model is shown with indication of ML bs and Bayesian pp values (pp values < 90 and bs values <50 are not shown); asterisks indicate full support in both the ML and Bayesian analyses.

A phylogeny based on currently available 18S rDNA sequences of Palmophyllales and Prasinococcales is shown in Fig. 6, and provides an indication of the known diversity within this group based on nuclear rDNA sequence data. The tree shows several well-supported clades that generally correspond to the two genera and three currently recognized species of Prasinococcales: *Prasinoderma coloniale*, *Prasinoderma singularis* and *Prasinococcus capsulatus*^37^,^38^,^39^. In addition, several clades represent undescribed diversity. DNA sequence data for the Palmophyllales are scarcer. Only three 18S rDNA sequences are currently available, representing the species *Palmophyllum umbracola* and *Verdigellas peltata*, which form a fully supported clade in the tree reported here. Only a few species have been described in the genera *Palmophyllum*, *Verdigellas* and *Palmoclathrus* (Supplementary Table S1), but sequence data from these different morphospecies and a wide geographical sampling will be needed to test generic boundaries and assess species diversity in the Palmophyllales. It is worth mentioning that genetic divergence between *Palmophyllum* and *Verdigellas* (max. p-distance 0.009) is much lower than between *Prasinoderma* and *Prasinococcus* (max. p-distance 0.108), or even between the two *Prasinoderma* species (max. p-distance 0.048).

### Evolution and systematics of the new class Palmophyllophyceae

Our phylogenetic and comparative genomic analyses provide compelling evidence that the Palmophyllales and Prasinococcales group in a distinct and well-supported clade that forms the deepest branch of the Chlorophyta.

The phylogenetic position of the Palmophyllales among the unicellular prasinophytes indicates an independent origin of macroscopic growth and multicellularity outside of the core Chlorophyta. Species of Palmophyllales form well-defined, attached macroscopic plants (thalli) composed of small, isolated, undifferentiated coccoid cells (3.2–10 μm) in a gelatinous matrix (palmelloid organisation)^22^,^23^,^24^. This type of macroscopic growth is rather atypical, as multicellularity in green algae usually involves cell–cell contact and cellular differentiation^40^. However, palmelloid thalli are found in a number of core Chlorophyta, including the Tetrasporales (Chlorophyceae), although they never form the elaborate large thalli found in the Palmophyllales^20^,^24^,^41^.

As discussed by Leliaert *et al.*^9^, the broad phylogenetic distribution of non-motile (coccoid) prasinophytes, including *Picocystis*, *Pycnococcus*, some species of Mamiellales, and the early-diverging Palmophyllales-Prasinococcales clade, may alter our understanding about the nature of the green plant ancestor. It is generally accepted that the ancestral green algae were unicellular flagellates (“ancestral green flagellate”) with characters typical of extant prasinophytes such as the presence of organic body scales^7^,^8^. Although it is indeed probable that flagella were present in a life cycle stage of the green plant ancestor, it is possible that this ancestor was a scale-less coccoid organism with transient flagellar stages^9^. Alternatively, coccoid forms may have evolved multiple times independently.

The sister relationship of the macroscopic Palmophyllales and the unicellular Prasinococcales is unusual from a morphological perspective, although, as will be discussed below, this relationship is supported by a number of shared cytological characteristics, such as cell size, lack of flagellar stages, presence of a mucus-secreting system, and similarities in cell division^21^,^37^,^39^,^41^,^42^. The morphological heterogeneity is not surprising given the large sequence distances within the clade, which likely reflects a great age of the divergences. Although dating the phylogeny of green plants is a difficult task because of the sparse fossil record of the group, our tentative time calibrated phylogeny (Supplementary Fig. S11) indeed suggests that the Palmophyllophyceae are ancient, having originated and diversified somewhere in the late Proterozoic and Paleozoic.

The Prasinococcales include only a few described species from marine environments, characterized by small (2.2–5.5 μm) coccoid, scale-less cells (Supplementary Table S1)^37^,^38^,^39^,^42^. Sexual reproduction has not been observed. Cells of *Prasinococcus* are typically embedded in gelatinous capsules secreted by complex pores (“Golgi-decapore complex”)^42^. *Prasinoderma* has a thick multi-layered cell wall without pores, and lacks a gelatinous envelope^39^. Traditionally, scale-less coccoid planktonic green algae were placed in the family Pycnococcaceae (Mamiellales), which initially included the genus *Pycnococcus*^43^, and subsequently *Prasinococcus* and *Prasinoderma*^37^,^38^. The grouping of *Prasinococcus* and *Prasinoderma* in a distinct clade (clade VI) separated from *Pycnococcus* (clade V) has been demonstrated by 18S rDNA phylogenetic data^10^,^11^.

The relationship of the Palmophyllales with the Prasinococcales is supported by a number of shared cytological features (Supplementary Table S1). Species of Palmophyllales and *Prasinococcus* both have a mucus-secreting system originating from a large Golgi body^21^. In *Prasinococcus*, the polysaccharide (mucus) capsule is secreted through a complex structure perforating the cell wall, which is composed of a round collared lid with 8 to 14 pores (“Golgi-decapore complex”)^42^. Species of Palmophyllales lack the complex decapore structure, and instead have simple pores in the cell wall^21^,^23^. Mode of cell division is also similar in species of Palmophyllales and Prasinococcales, characterized by unequal binary fission. In *Prasinococcus* and *Prasinoderma*, one of the daughter cells retains the parent wall, while the other is released with a newly produced cell wall^37^,^38^,^39^. In *Palmoclathrus* (the only species of Palmophyllales where cell division has been studied in detail), the parental cell wall is discarded and incorporated into the gelatinous matrix^41^. Finally, cells of Palmophyllales and Prasinococcales lack flagella or ultrastructural traces from flagella (basal bodies, centrioles)^21^,^23^,^41^. This feature, however, is not unique to the clade.

Taken together, the phylogenetic distinctness of the Palmophyllales-Prasinococcales clade, and the presence of some unique phenotypic features warrant recognition of a new class of Chlorophyta. In the currently accepted classification of the Viridiplantae, the major clades of the Streptophyta and core Chlorophyta are classified at the class level, as are some of the major prasinophyte clades, including Nephroselmidophyceae and Mamiellophyceae^1^,^14^,^44^. Our proposal for a new class entirely fits this taxonomic scheme.

### Class Palmophyllophyceae Leliaert *et al.* class. nov.

#### Description

Marine green algae. Cells planktonic, solitary or in loose colonies, or cells grouped in a gelatinous matrix forming benthic macroscopic thalli. Cells spherical or subspherical, lacking flagella and organic body scales, with a single cup-shaped chloroplast enclosing a mitochondrion, nucleus, and large Golgi body. Cell surrounded by a cell wall, with or without pores. Chloroplast surrounded by two membranes, with chlorophylls *a* and *b*, with or without pyrenoid. Cell division by unequal binary fission. Strongly supported clade in plastid multi-gene and nuclear ribosomal DNA phylogenetic analyses.

Order Palmophyllales Zechman *et al.*^20^.

Family Palmophyllaceae Zechman *et al.*^20^.

Genera *Palmophyllum* Kützing (type genus), *Verdigellas* D.L. Ballantine & J.N. Norris, *Palmoclathrus* Womersley.

Order Prasinococcales Guillou *et al.*^11^.

#### Description

Marine planktonic green algae. Cells solitary or forming loose colonies. Cells spherical or subspherical, lacking flagella and organic body scales, with a thin cell wall surrounded by a thick ellipsoidal gelatinous capsule, or with a thick, multi-layered cell wall without gelatinous capsule. Cells with a single cup-shaped chloroplast enclosing a mitochondrion, nucleus, and large Golgi body. Chloroplast with a large pyrenoid surrounded by a starch sheath; pyrenoid matrix penetrated by a bifurcate extension of the cytoplasm and the mitochondrion. Cell division by unequal binary fission in which one of the daughter cells retains the parent wall, while the other is released with a newly produced cell wall. Main pigments include chlorophylls *a* and *b*, prasinoxanthin, Mg-2,4-divinylphaeoporphyrin a5 monomethylester (MgDVP), uriolide, and micromonol.

Family Prasinococcaceae Leliaert fam. nov.

Characters as for order.

Genera *Prasinococcus* H. Miyashita & M. Chihara (type genus) and *Prasinoderma* T. Hasegawa & M. Chihara.

#### Nomenclatural notes

The order Prasinococcales was originally described by Chadefaud^45^ for the single species *Halosphaera viridis* (descriptive order name according to article 16.1 of the International Code of Nomenclature (ICN)^46^). Since *Halosphaera* is now considered a member of the Pyramimonadales^7^,^13^,^47^, Prasinococcales Chadefaud is a synonym of Pyramimonadales. More recently, Guillou *et al.*^11^ used the name Prasinococcales to label “prasinophyte clade VI”^10^, which includes *Prasinococcus* (Miyashita *et al.* 1993)^38^ and *Prasinoderma* (Hasegawa *et al.* 1996)^37^. In the interpretation of Guillou *et al.*^11^, which is different from Chadefaud, Prasinococcales is an automatically typified name according to article 16.1 of the ICN, with type *Prasinococcus*. Because Guillou *et al.*^11^ did not provide a description for the order, we provide one here. Although the family Prasinococcaceae is flagged as an accepted family name in the Global Biodiversity Information Facility (GBIF: www.gbif.org) and in AlgaeBase (algaebase.org), the name has never been described nor validly published, hence the formal description in this paper.

## Conclusion

We provide solid phylogenetic evidence that the enigmatic Palmophyllales together with the Prasinococcales form the deepest-branching clade of the Chlorophyta, which we describe as a new class, the Palmophyllophyceae. Our phylogenetic results improve our understanding of morphological evolution in the green plants. Until present, the early-diverging lineages of the Chlorophyta (the prasinophytes) were only known to comprise unicellular planktonic algae. Our results point to an independent origin of macroscopic growth and multicellularity outside of the core Chlorophyta. Our study also contributes to a better understanding of plastid genome evolution in green plants. The small, compact and intronless cpDNA of *Verdigellas peltata* shows remarkable similarities in gene content and organization with the cpDNAs of Prasinococcales and the streptophyte *Mesostigma viride*, indicating that cpDNA architecture has been extremely well conserved in the early-branching lineages of green plants.

## Methods

### Sampling

Material of *Verdigellas peltata* was obtained from a dredged sample offshore Louisiana, Ewing Bank, Gulf of Mexico, at ca. 70 m depth (Supplementary Fig. S1). A voucher specimen was deposited in the Herbarium of the University of Louisiana at Lafayette (LAF-8-26-12-6-1), and a portion of the specimen was dried in silica gel for molecular analysis. The specimen was morphologically identified as *Verdigellas peltata*, and DNA-confirmed based on the chloroplast *rbc*L (NCBI-megablast: 98.8% identity with *V. peltata*, GenBank accession: EU586183) and nuclear 18S rRNA genes (99.7% identity with *V. peltata*, GenBank accession: FJ619277), which were the only sequences publicly available for *V. peltata* previous to this study.

### Sequencing, assembly and annotation of the chloroplast genome and nuclear rDNA cistron

Total genomic DNA was extracted using the E.Z.N.A. Plant DNA Kit (OMEGA Bio-tek, Norcross, GA, USA). The sequencing library was prepared using the Nextera DNA Sample Prep Kit (Illumina, San Diego, CA, USA). Sequencing was performed using Illumina MiSeq technology, generating 5.9 million paired-end reads of 2 × 250 bp and 6.6 million paired-end reads of 2 × 150 bp. Low-quality ends of the reads (Phred score <30) and adapters were trimmed using Trim Galore! (www.bioinformatics.babraham.ac.uk/projects/trim_galore). *De novo* assembly of paired-end reads was performed using Velvet v. 1.2.10^48^ and a *k*-mer length of 91, as well as the CLC Genomics Workbench (CLC Bio, Aarhus, Denmark) *de novo* assembler with default parameters (word size = 23). Contiguous DNA sequences (contigs) of the chloroplast genome were identified by blastn similarity searches (E-value <10^−6^) against a custom-built database of 1305 gene sequences from published cpDNAs of green algae. Raw reads were iteratively mapped (10×) to these putative cpDNA contigs under stringent conditions (no gaps allowed, minimum overlap of 25 nucleotides, and minimum overlap identity of 98%) in Geneious (Biomatters, www.geneious.com). The resulting extended contigs were visually examined, and consensus sequences re-assembled to obtain a large scaffold that could be closed into a circle by an overlap of 456 bp. The assembly had an average coverage of 28× (min. 4×, max. 67×). The sequence with 4× coverage was 9 bp long and situated between the *trnR*(ucu) and *chlI* genes.

Genes were initially identified by mapping the abovementioned 1305 green algal chloroplast gene sequences against the cpDNA contigs using the read mapper in Geneious. The annotations were verified by identifying ORFs in Geneious, followed by blastp searches against the NCBI nonredundant database (http://blast.ncbi.nlm.nih.gov/Blast.cgi, last accessed December 7, 2015). The boundaries of the rRNA genes were identified based on a dataset of aligned complete rRNA genes from published green algal cpDNAs. tRNA genes were detected and identified using tRNAscan-SE 1.21^49^. The circular genome map was drawn with OGDRAW^50^.

The contig containing the nuclear-encoded rDNA cistron was identified by blastn similarity search of a custom-built blast database of complete small and large subunit rRNA genes from green algae. The boundaries of the rRNA genes were identified based on an alignment of published complete rRNA genes.

### Comparative analyses of chloroplast gene order

A custom-built program was used to identify syntenic regions between the chloroplast genomes of *Verdigellas* and early-diverging green algae. This program was also employed to convert gene order in each of 15 selected green algal cpDNAs to all possible pairs of signed genes (i.e. taking into account gene polarity). The presence/absence of the signed gene pairs in three or more genomes were coded as binary characters using Mesquite 3.04^51^.

### Phylogenetic analyses

Phylogenetic analyses were based on four datasets. Gene and protein alignments were produced from 71 chloroplast genes and their predicted protein products; these sequences were obtained from complete (or in few cases partial) chloroplast genomes of 59 taxa of Archaeplastida, including 51 Viridiplantae and eight outgroup taxa (seven Rhodophyta and one Glaucophyta). A third alignment consisted of (near) complete sequences for the nuclear small (18S) and large (28S) subunit rRNA genes of 63 Viridiplantae and three outgroup taxa. A fourth alignment consisted of 18S rDNA sequences from 27 taxa of Prasinococcales and Palmophyllales, and four prasinophyte outgroup taxa. Taxon lists are provided in Supplementary Tables S2 and S3.

Taxon and gene sampling for the 71-chloroplast gene and protein alignments was largely based on Lemieux *et al.*^16^, with selected genes: *accD, atpA, B, E, F, H, I, ccsA, cemA, chlB, I, L, N, P, ftsH, infA, petA, B, D, G, L, psaA, B, C, I, J, M, psbA, B, C, D, E, F, H, I, J, K, L, M, N, T, Z, rbcL, rpl2, 5, 14, 16, 20, 23, 32, 36, rpoA, B, C1, C2, rps2, 3, 4, 7, 8, 9, 11, 12, 14, 18, 19, tufA, ycf1,3,4,12*. The dataset was 71% filled at the taxon × gene level. DNA sequences were aligned for each gene separately using the ClustalW translational alignment function^52^ in Geneious with a BLOSUM cost matrix, a gap open penalty of 10, and a gap extension cost of 0.1. The separate gene alignments were then concatenated, and poorly aligned codons removed using the Gblocks server^53^ (http://molevol.cmima.csic.es/castresana/Gblocks_server.html) and the least stringent settings, allowing smaller final blocks, gap positions within the final blocks, less strict flanking positions and many contiguous non-conserved positions. Gblocks reduced the alignment from 146,202 to 44,493 positions. Only the first two codon positions in the nucleotide alignment were included for phylogenetic analyses (29,662 positions). Gblocks with the least stringent settings was also applied to the protein alignment, reducing the alignment from 48,759 to 13,730 amino acid positions.

Taxon and gene sampling for the nuclear rDNA alignment was based on Marin^18^ and extended with species of Streptophyta, Rhodophyta and Glaucophyta for which (near) complete small (18S) and large (28S) subunit rRNA genes were available. 18S and 28S rDNA sequences were aligned separately using MUSCLE^54^. Both alignments (1876 and 3501 positions, respectively) were concatenated, and poorly aligned positions removed using the Gblocks with the least stringent settings (see above). Gblocks reduced the alignment from 5377 to 4579 positions.

Plastid phylogenies were inferred from the amino acid and nucleotide datasets using Bayesian and ML analyses. For the amino acid dataset, the cpREV + Γ4 + F model of evolution was selected as the best-fitting model using ProtTest 3.2^55^ based on the corrected Akaike Information Criterion. For the nucleotide dataset, we selected the GTR + Γ4 + I model and a partitioning strategy in which codon positions were treated separately (2 partitions) based on the outcome of the BIC criterion in PartitionFinder^56^. Bayesian analyses of both the amino acid and nucleotide datasets were conducted using MrBayes v.3.2.1^57^ with a single partition for the amino acid analysis, and two partitions with unlinked models for the nucleotide analysis. Two independent runs were performed using 1 million (amino acid dataset) or 3 million (nucleotide dataset) generations, each with one cold and three heated chains, and sampling every 1000 generations. The first 20% (amino acid analysis) or 10% (nucleotide analysis) of samples were discarded as burn-in based on assessment of convergence of the runs and stability of parameters using Tracer v.1.5^58^. ML trees were inferred using RAxML v. 8.2.4^59^ using the same partitioning strategies, and bootstrapping with 500 replicates to assess branch support.

In addition, the amino acid dataset was analysed using the site-heterogeneous CAT + Γ4 and CATGTR + Γ4 models of evolution with PhyloBayes v. 4.1^60^,^61^. Five independent chains were run for 10,000 cycles for the CAT + Γ4 analysis and 2,000 cycles for the CATGTR + Γ4 analysis and a consensus topology was calculated from the saved trees using the BPCOMP program of PhyloBayes after a burn-in of 2,000 and 500 cycles, respectively. A maxdiff of 1 was obtained in both analyses, indicating that at least one of the runs was stuck in a local maximum.

We also addressed possible phylogenetic artefacts due to potential biases in amino acid composition resulting from the low GC content in the cpDNAs of *Verdigellas* and clade VI prasinophytes^27^,^62^. In order to overcome non-phylogenetic signal due to compositional heterogeneity, we performed a phylogenetic analysis based on a Dayhoff recoded dataset^63^,^64^ using PhyloBayes with a homogeneous GTR + Γ4 model of evolution. The amino acid data was recoded using the -recode option of PhyloBayes and the following Dayhoff6 recoding scheme: (A,G,P,S,T) (D,E,N,Q) (H,K,R) (F,Y,W) (I,L,M,V) (C). Five independent chains were run for 3,000 cycles and a consensus topology was calculated after a burn-in of 600 cycles.

A provisional time-calibrated phylogeny was obtained with BEAST v. 1.8.2^65^ with two nodes constrained in time based on previous molecular clock analyses^1^: The root of the Viridiplantae was constrained using a normal prior with mean 970 Mya, standard deviation 200, and a minimum and maximum age of 655 and 1280 Mya, respectively; the root of the land plants was constrained using a normal prior with mean 475 Mya and standard deviation 20. Details of the BEAST analysis are provided in Supplementary Fig. 11. Because the root age of the Viridiplantae is highly uncertain, these results should be regarded as tentative.

Nuclear rDNA-based phylogenies were inferred using unlinked GTR + Γ4 + I models for the 18S and 28S partitions selected based on the BIC criterion in PartitionFinder. MrBayes analyses included two independent runs of 5 million generations (each with four chains). The first 10% of samples were discarded as burn-in. ML trees were inferred using RAxML v. 8.2.4 using the same partitioning strategy, and bootstrapping with 500 replicates to assess branch support.

The nuclear 18S rDNA dataset of Prasinococcales and Palmophyllales was based on Viprey *et al.*^66^ and Jouenne *et al.*^39^ and extended with sequences retrieved from blastn searches of *Prasinococcus*, *Prasinoderma* and *Verdigellas* 18S sequences. Phylogenetic analyses of this dataset (1778 positions) was performed under the GTR + Γ4 + I model with MrBayes (2 million generations, two runs of four chains each, and the first 500 thousand samples discarded as burnin) and RAxML with default settings.

Phylogenetic analyses were run on the CIPRES Science Gateway v3.3^67^ and Katak server of the Institut de Biologie Intégrative et des Systèmes of Université Laval (http://www.ibis.ulaval.ca/?pg=bioinformatique_accesServeurs).

## Additional Information

**Accession numbers**: The complete chloroplast genome and nuclear ribosomal DNA unit of Verdigellas peltata have been deposited in the European Nucleotide Archive with INSDC (GenBank, EMBL-EBI/ENA, DDBJ) accession numbers LT174527 and LT174528, respectively. Sequence alignments and trees are available from TreeBase (study ID 19074).

**How to cite this article**: Leliaert, F. *et al.* Chloroplast phylogenomic analyses reveal the deepest-branching lineage of the Chlorophyta, Palmophyllophyceae class. nov. *Sci. Rep.* **6**, 25367; doi: 10.1038/srep25367 (2016).

## Supplementary Material

Supplementary Information
