# Supplementary material for: Chloroplast phylogenomic analyses reveal the deepest-branching lineage of the Chlorophyta, Palmophyllophyceae class. nov
Source: Sci Rep. 2016 May 9;6:25367. doi: 10.1038/srep25367 (PMC4860620; doi:10.1038/srep25367)
Supplement: Supplementary Information [file srep25367-s1.pdf]

Supplementary Information

Chloroplast phylogenomic analyses reveal the deepest-branching lineage of the Chlorophyta, Palmophyllophyceae class. nov.

Frederik Leliaert, Ana Tronholm, Claude Lemieux, Monique Turmel, Michael S. DePriest, Debashish Bhattacharya, Kenneth G. Karol, Suzanne Fredericq, Frederick W. Zechman, and Juan M. Lopez-Bautista

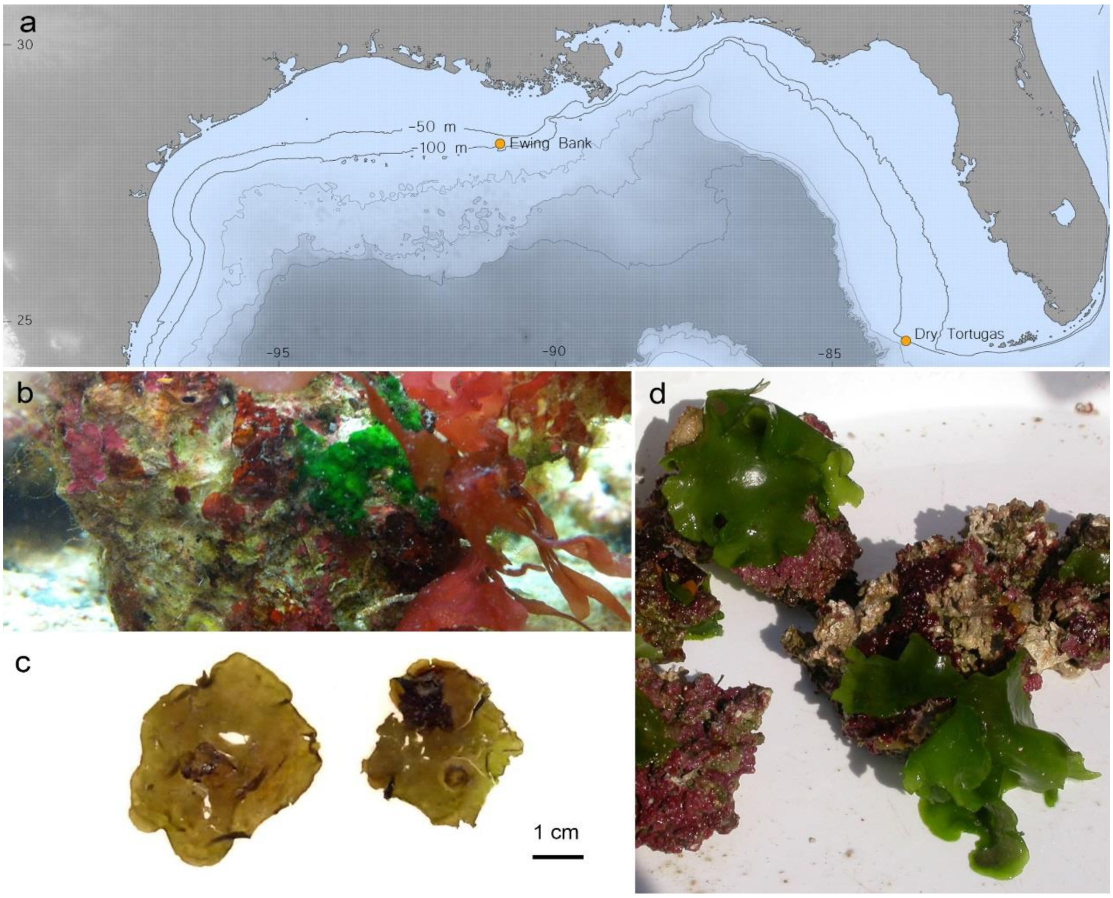

Figure S1. (a) Location of the sequenced specimen of *Verdigellas peltata*: offshore Louisiana, Ewing Bank, NW Gulf of Mexico (box dredge in: 28 05.58'N, 91 01.01'W; box dredge out: 28 05 67'N, 91 01.49'W), at ca. 70 m depth, and the specimen depicted in Fig. S1d. Map created using the R [1] package marmap v. 0.9.5 [2] (https://cran.r-project.org/web/packages/marmap) **(b)** Photograph of live specimen, maintained in laboratory microcosm, growing from surface of rhodolith (LAF-8-26-12-6-1). **(c)** Photograph of the herbarium voucher of the sequenced specimen (LAF-8-26-12-6-1). **(d)** *Verdigellas peltata* collected in the vicinity of Dry Tortugas, FL, SE Gulf of Mexico (2 Jun 2004, 24°39.03'N, 83°41.02'W, growing on rhodoliths among red algal crusts, depth between 104 and 94 m, collected with box dredge aboard the R/V Pelican, coll. S. Fredericq (NSF-I-30-1, LAF-6-2-04-3-1).

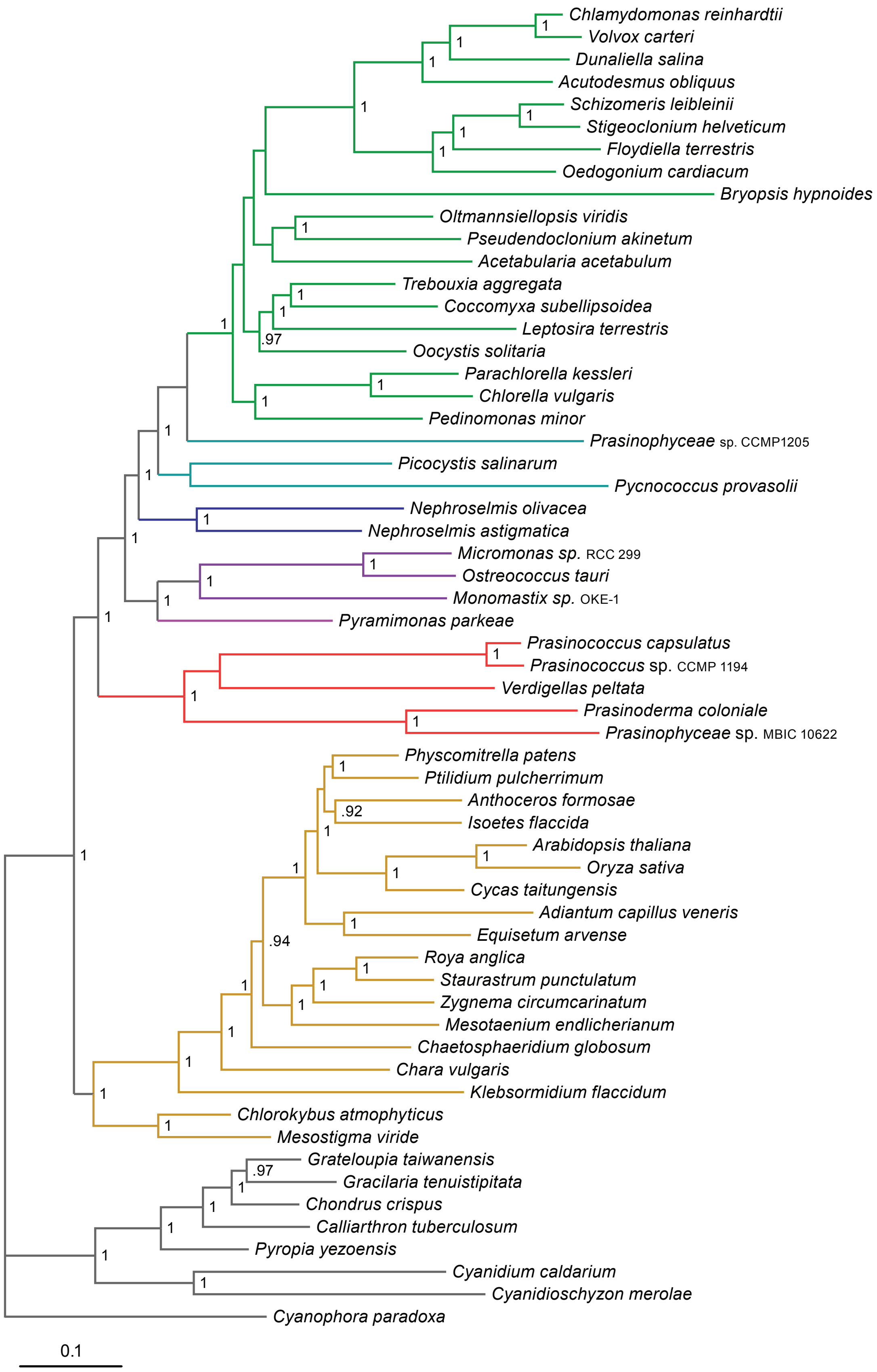

Figure S2. Plastid phylogeny of the green plants obtained by Bayesian inference (MrBayes) of a concatenated protein alignment of 71 chloroplast genes (13,730 amino acid positions) under a cpREV+Γ4+F model. The majority rule consensus tree is shown with node support given as posterior probabilities (values < .90 are not shown).

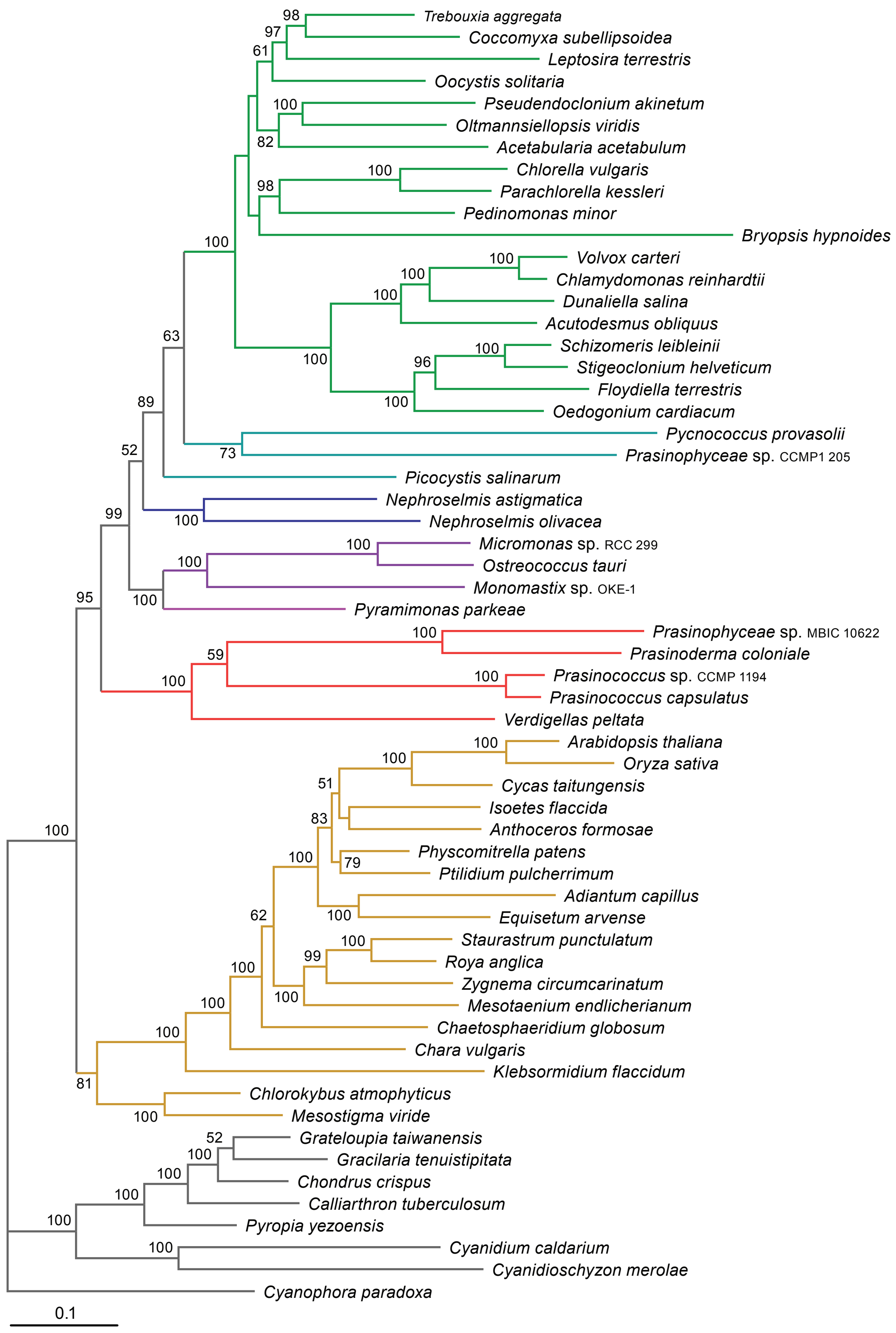

Figure S3. Plastid phylogeny of the green plants obtained by maximum likelihood inference (RAxML) of a concatenated protein alignment of 71 chloroplast genes (13,730 amino acid positions) under a cpREV+Γ4+F model. The maximum likelihood tree is shown with bootstrap support for the nodes (values < 50 are not shown).

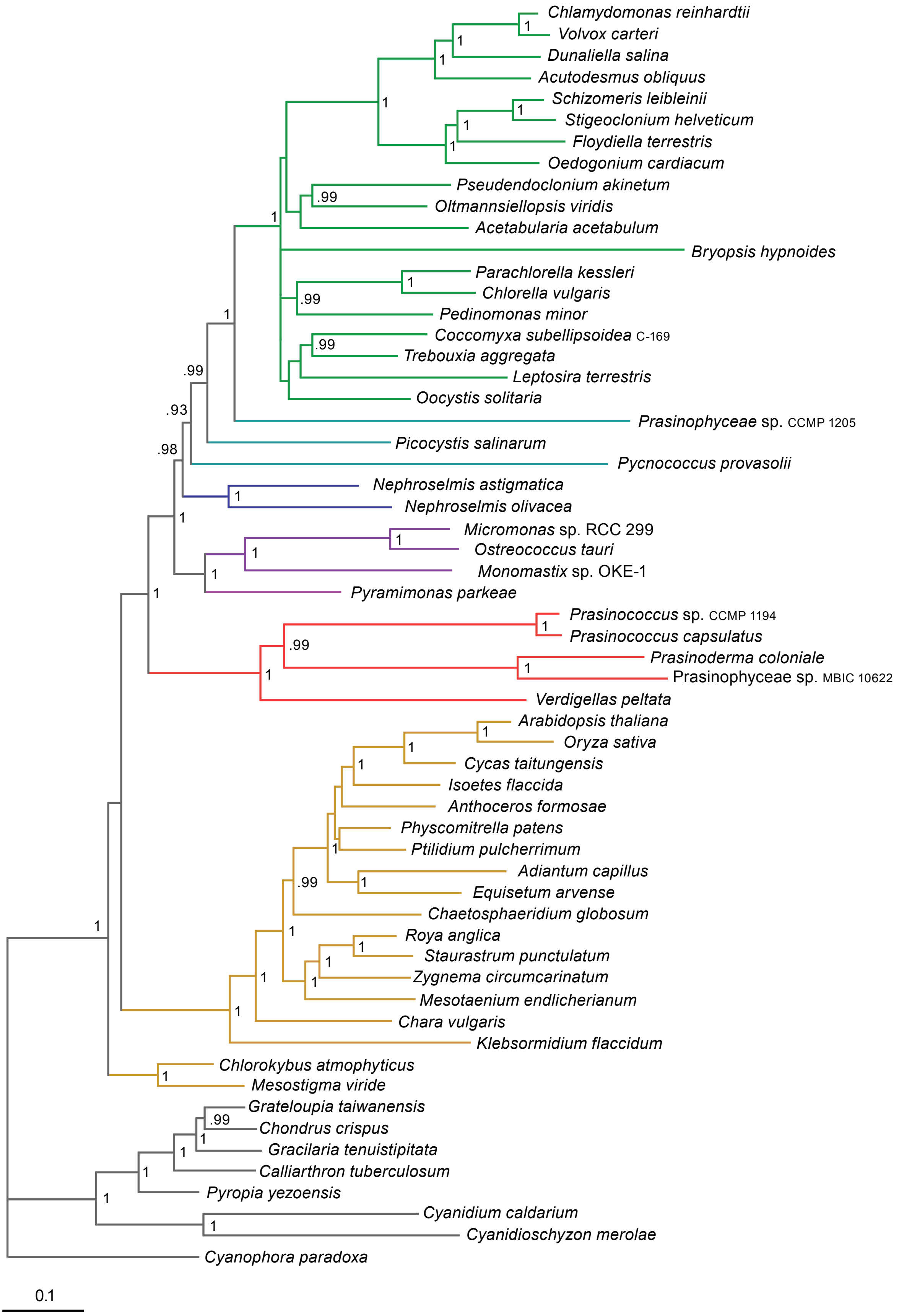

Figure S4. Plastid phylogeny of the green plants obtained by Bayesian inference (Phylobayes) of a concatenated protein alignment of 71 chloroplast genes (13,730 amino acid positions) under a site-heterogeneous CAT+Γ4 model. The consensus tree is shown with node support given as posterior probabilities (values < .90 are not shown).

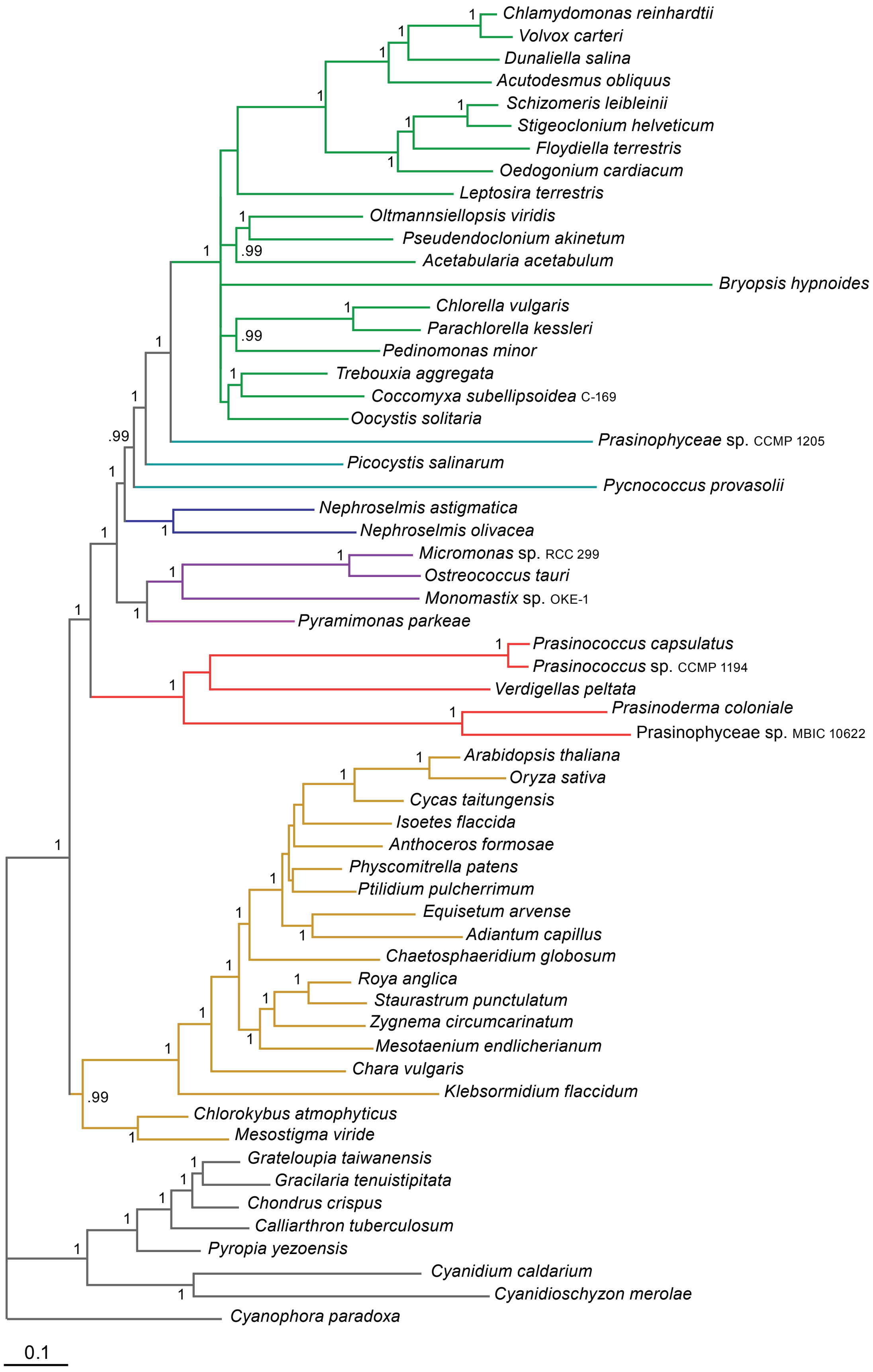

Figure S5. Plastid phylogeny of the green plants obtained by Bayesian inference (Phylobayes) of a concatenated protein alignment of 71 chloroplast genes (13,730 amino acid positions) under a site-heterogeneous CATGTR+Γ4 model. The consensus tree is shown with node support given as posterior probabilities (values < .90 are not shown).

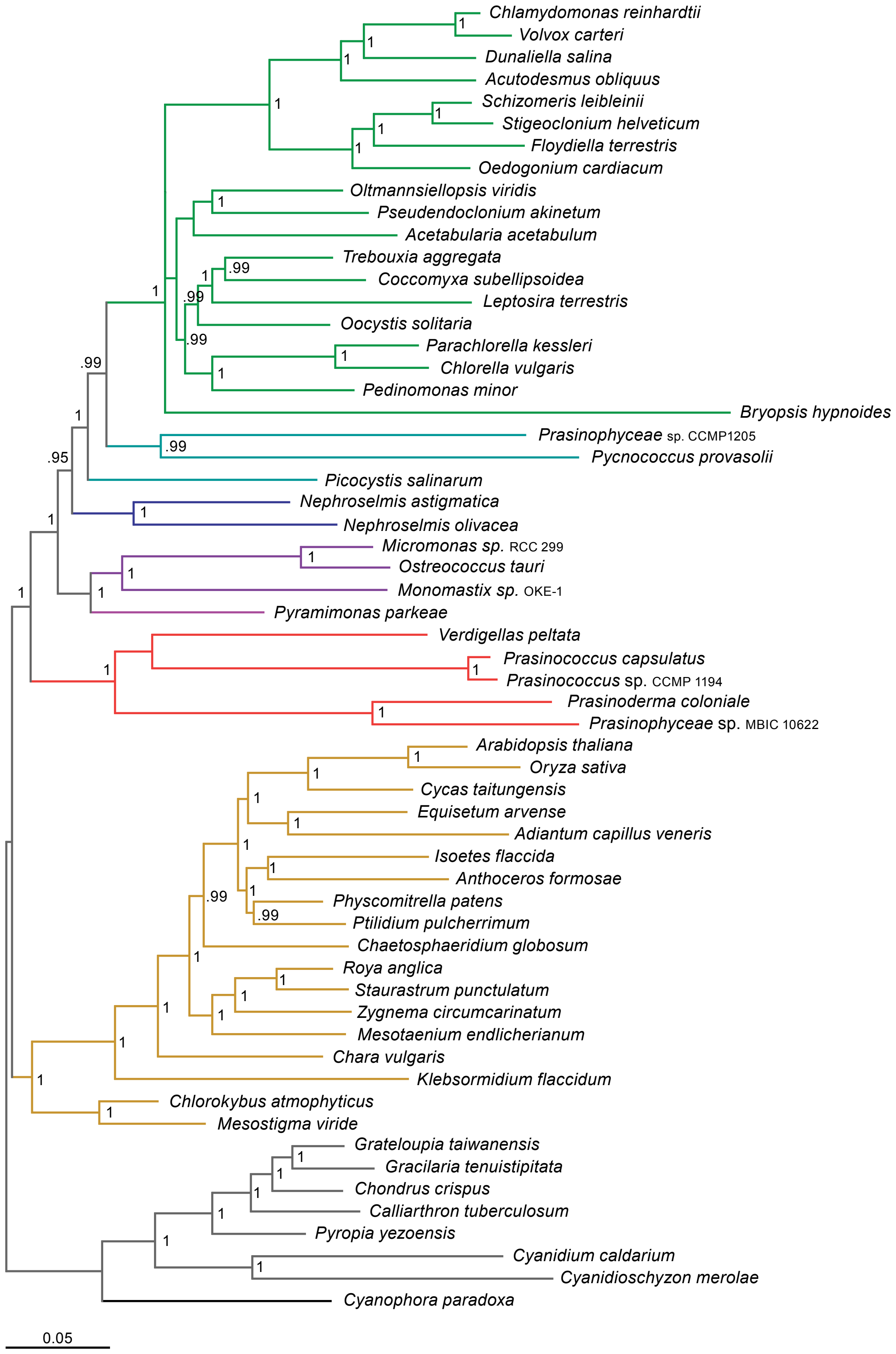

Figure S6. Plastid phylogeny of the green plants obtained by Bayesian inference (Phylobayes) of the concatenated protein alignment of 71 chloroplast genes (13,730 amino acid positions), recoded by the Dayhoff6 recoding scheme, and analysed under a GTR+Γ4 model. The consensus tree is shown with node support given as posterior probabilities (values < .90 are not shown)

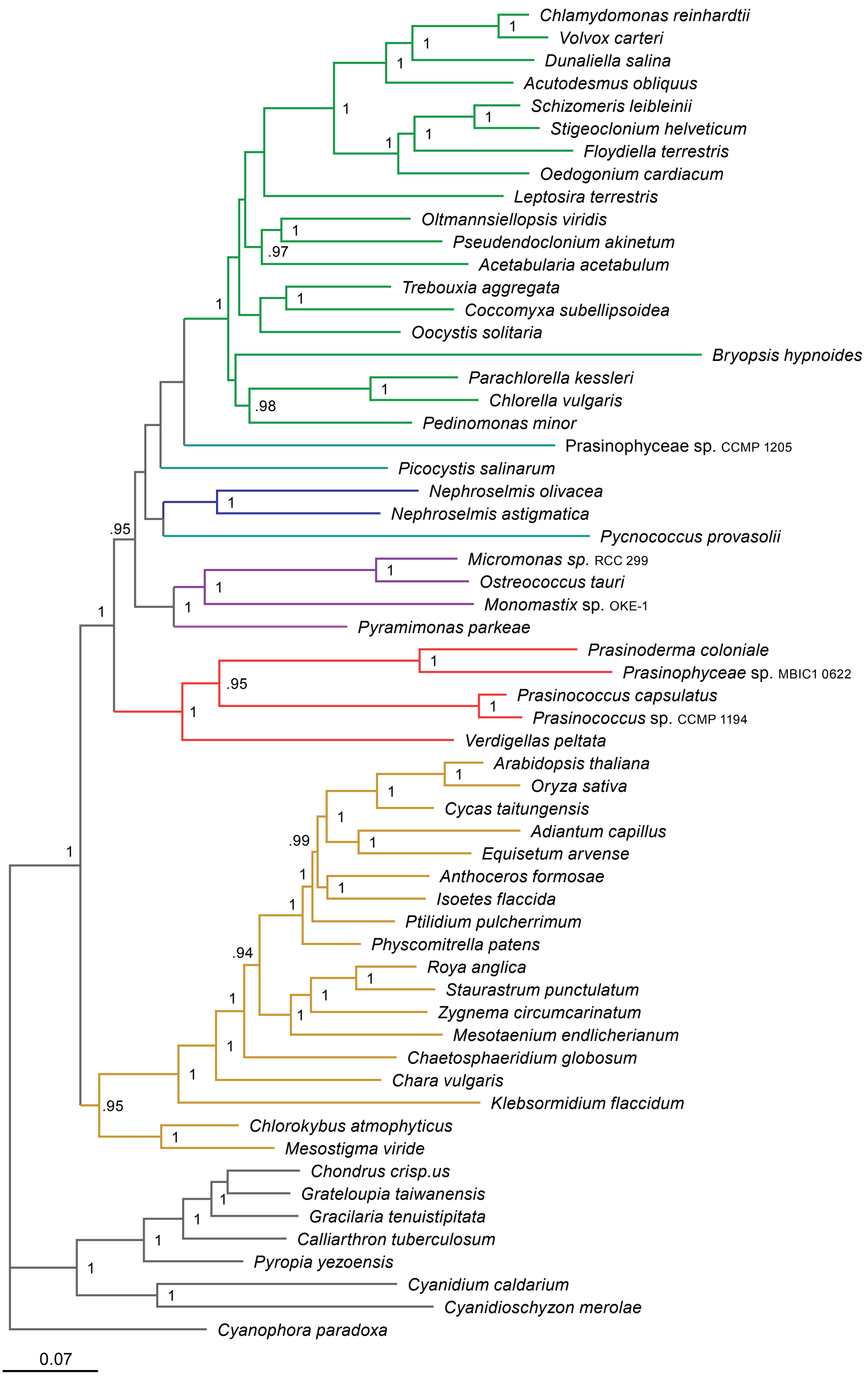

Figure S7. Plastid phylogeny of the green plants obtained by Bayesian inference (MrBayes) of a concatenated nucleotide alignment of 71 chloroplast genes (1st and 2nd codon position: 29,662 positions) under a GTR+Γ4+I model and a partitioning strategy in which codon positions were treated separately (2 partitions). The majority rule consensus tree is shown with node support given as posterior probabilities (values < .90 are not shown).

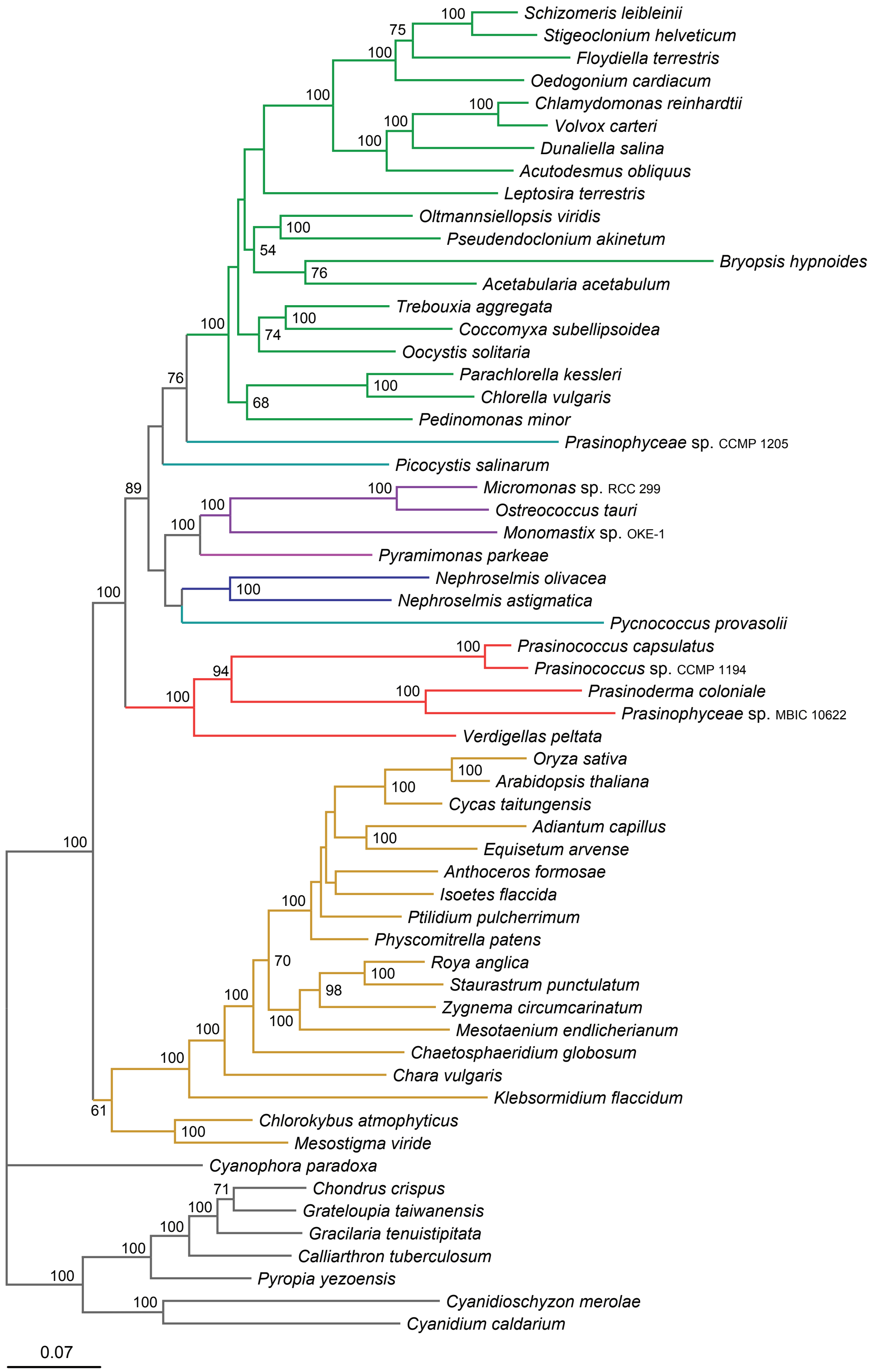

Figure S8. Plastid phylogeny of the green plants obtained by maximum likelihood inference (RAxML) of a concatenated nucleotide alignment of 71 chloroplast genes (1st and 2nd codon position: 29,662 positions) under a GTR+Γ4+I model and a partitioning strategy in which codon positions were treated separately (2 partitions). The maximum likelihood tree is shown with bootstrap support for the nodes (values < 50 are not shown).

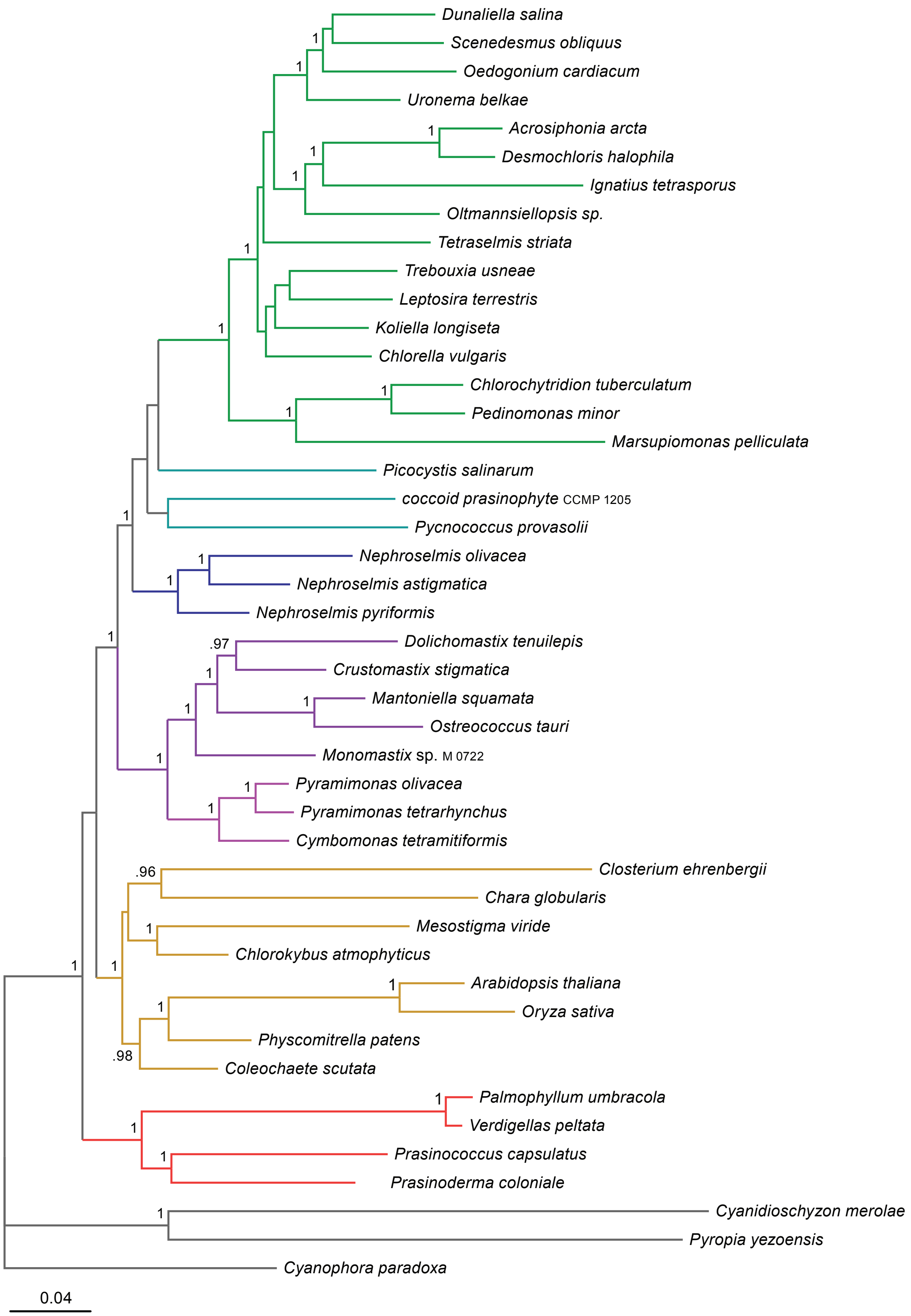

Figure S9. Nuclear rDNA phylogeny of the green plants obtained by Bayesian inference (MrBayes) of a concatenated alignment of 18S and 28S rRNA genes (4,579 positions) using unlinked GTR+Γ4+I models for the 18S and 28S partitions. The majority rule consensus tree is shown with node support given as posterior probabilities (values < .90 are not shown).

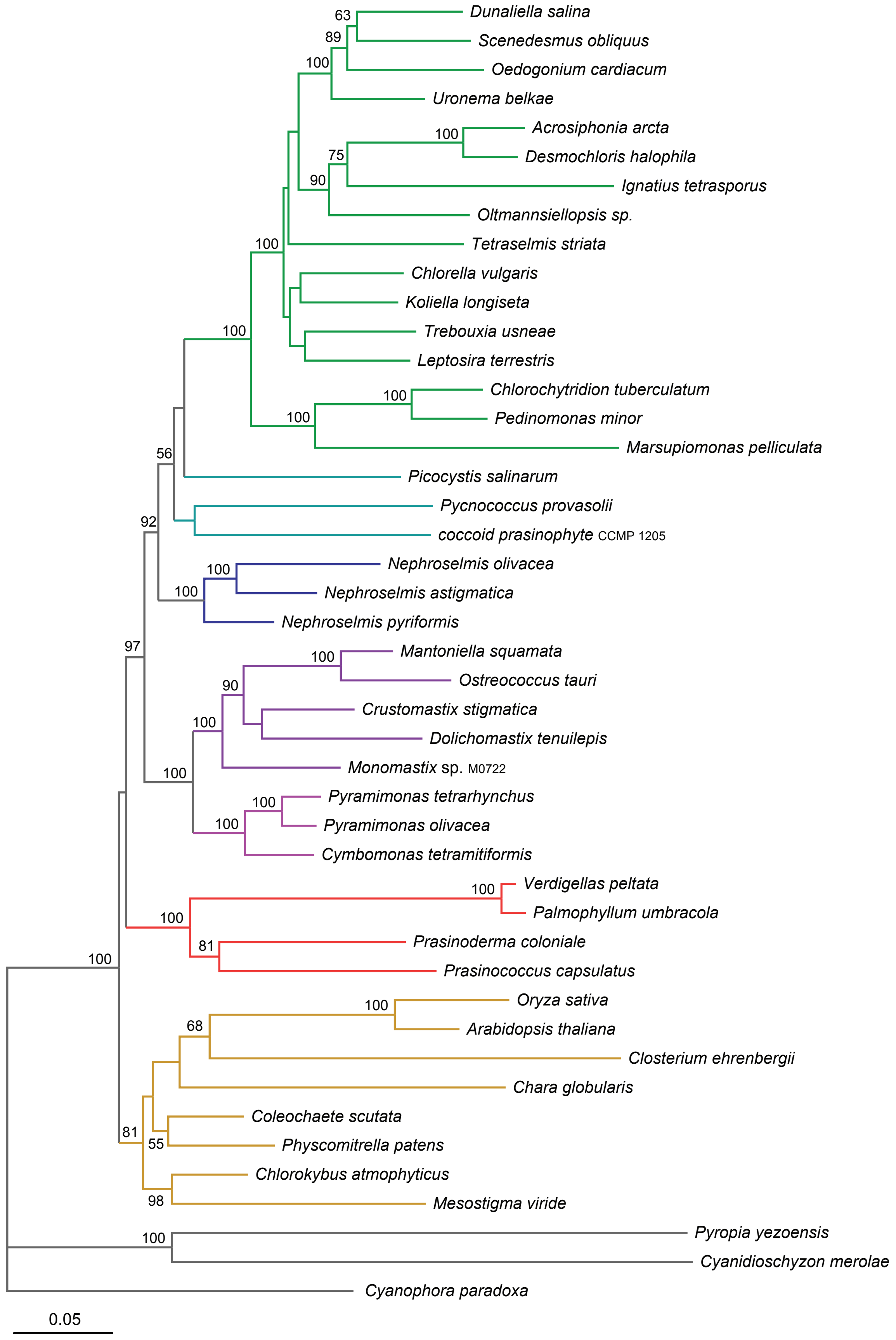

Figure S10. Nuclear rDNA phylogeny of the green plants obtained by maximum likelihood inference (RAxML) of a concatenated alignment of 18S and 28S rRNA genes (4,579 positions) using unlinked GTR+Γ4+I models for the 18S and 28S partitions. The maximum likelihood tree is shown with bootstrap support for the nodes (values < 50 are not shown).

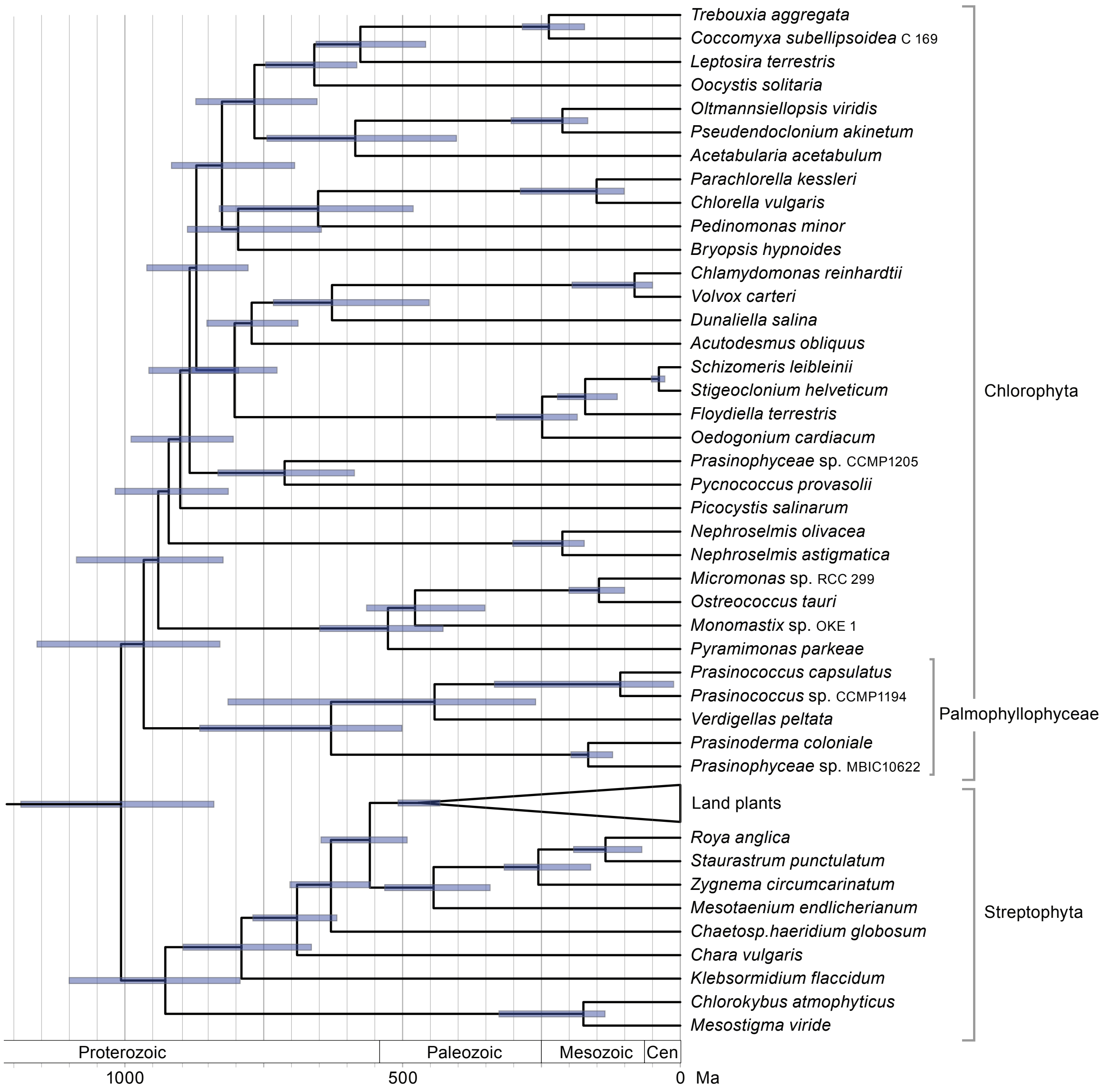

Figure S11. Tentative time-calibrated phylogeny of the green plants obtained by Bayesian inference with BEAST v1.8.2 [3,4] based on a concatenated protein alignment of 71 chloroplast genes (13,730 amino acid positions), using a cpREV+Γ4 model with divergence times estimated under an uncorrelated lognormal relaxed molecular clock model and the birth-death tree prior. The majority rule consensus tree obtained from the MrBayes analysis using a cpREV+ Γ4+F model (Figs 4, S2) was used to fix the topolgy. Two nodes in the tree were constrained in time based on previous molecular clock analyses: The root of the Viridiplantae was constrained using a normal prior with mean 970 Mya, standard deviation 200, and truncated at 655 and 1280 Mya based on previous studies [5-11]. The root of the land plants was constrained using a normal prior with mean 475 Mya and standard deviation 20 [12]. Markov Chain Monte Carlo (MCMC) analyses were run for 10 million generations, with subsampling every 1,000 generations, and summary statistics and trees were generated using the last 5 million generations. Outgroups have been pruned from the tree after the analysis.

Table S1. Comparison of phenotypic features between genera of the Palmophyllophyceae class. nov.

Genus	***Prasinococcus***	***Prasinoderma***	***Verdigellas***	***Palmophyllum***	***Palmoclathrus***
Currently accepted species [13]	*P. capsulatu*	*P. coloniale*, *P. singularis*	*V. fimbriata*, *V. peltata V. nektongammea*	*P. crassum*, *P. umbracola*	*P. stipitatus*
Thallus morphology	Unicellular; cells solitary.	Unicellular; cells solitary (*P. singularis*) or forming loose colonies (*P. coloniale*).	Thallus macroscopic, composed of isolated spherical cells in a gelatinous matrix.<br>Thallus with one or several stipes and blades of various form.	Thallus macroscopic, composed of isolated spherical cells in a gelatinous matrix.<br>Thallus irregular crustose lobes.	Thallus macroscopic, composed of isolated spherical cells in a gelatinous matrix.<br>Thallus with a distinctive stipe and a cup-shaped, perforate blade.
Cell morphology	Coccoid, (sub)spherical, 3.5-5.5 (-8) µm long.	Coccoid, spherical, 2.2-5.5 µm in diameter.	Coccoid, (sub)spherical, 3.2-10 µm long.	Coccoid, subspherical, 6-7 µm long.	Coccoid, subspherical, 8-10 µm long.
Cell wall	Thin cell wall surrounded by a thick ellipsoidal gelatinous capsule (including the polysaccharide capsulan).	Thick, multi-layered cell wall, sometimes surrounded by a mucus-like secretion (*P. singularis*).	Thin cell wall with extended external gelatinous matrix forming the thallus. Cell walls thickened by trilaminar sheets to inner surface.	Thin cell wall with extended external gelatinous matrix forming the thallus.	Thin cell wall with extended external gelatinous matrix forming the thallus.
Subcellular characteristics	Cup-shaped chloroplast enclosing a single mitochondrion, a nucleus, and a large Golgi body.<br>Chloroplast with a large pyrenoid surrounded by a starch sheath; pyrenoid matrix penetrated by a bifurcate extension of the cytoplasm and the mitochondrion. [a]<br>Cell wall at the open end of the cup-shaped chloroplast perforated by a complex structure composed of a round collared lid, with 8 to 14 pores ("decapore structure"). [b]	Cup-shaped chloroplast enclosing a single mitochondrion, a nucleus, and a large Golgi body.<br>Chloroplast with a large pyrenoid surrounded by a starch sheath; pyrenoid matrix penetrated by a bifurcate extension of the cytoplasm and the mitochondrion. [a]<br>Cell wall lacking pores or projecting appendages.	Cup-shaped chloroplast enclosing a nucleus, a large Golgi body, a large vacuole, and an inconspicuous mitochondrion.<br>Chloroplast with scattered starch grains; without pyrenoid.<br>Vacuole with heterogeneous content, including amorphous, fibrillar, and trilaminar components.<br>Cell wall at the open end of the cup-shaped chloroplast perforated by pores.<br>Lumen of the nuclear envelope with spherical glycoprotein inclusions.	Cup-shaped chloroplast enclosing a nucleus, a large Golgi body (sometimes 2 or 3), a large vacuole ("dictyosome-associated membrane-bound body"), and a mitochondrion.<br>Chloroplast with scattered starch grains; without pyrenoid.<br>Cell wall at the open end of the cup-shaped chloroplast perforated by pores ("mucilage secretion organelles").	Cup-shaped chloroplast enclosing a nucleus, a large Golgi body, a large dictyosome-associated vacuole ("membrane bound body"), and a mitochondrion.<br>Chloroplast with scattered starch grains; without pyrenoid.
Cell division	Unequal binary fission in which one of the daughter cells retains the parent wall, while the other is released with a newly produced cell wall.	Unequal binary fission in which one of the daughter cells retains the parent wall, while the other is released with a newly produced cell wall.	Not studied.	Not studied.	Unequal binary fission associated with the segregation of the prominent membrane-bound body into only one of the two daughter cells. Parental cell wall is discarded and incorporated into the gelatinous matrix.
Major pigments	Chlorophylls a and b, prasinoxanthin, MgDVP, uriolide, micromonol; chlorophyll b/a ratio ~ 0.6-1. [c]	Chlorophylls a and b, prasinoxanthin, MgDVP, uriolide, micromonol; chlorophyll b/a ratio ~ 0.6-0.9. [c]	Not studied.	Chlorophylls a and b, carotene and xanthophylls including neoxanthin as well as an unidentified orange pigment; chlorophyll b/a ratio ~ 1.6. [d]	Chlorophylls a and b, carotene, and xanthophylls including lutein, violaxanthin and neoxanthin; chlorophyll b/a ratio ~ 1. [d]
Habitat	Coastal to oceanic waters from surface to 200 m deep.	Coastal to oceanic waters, from surface to -40 m deep	Benthic, locally abundant on deep-reef slopes, 40-150 m deep.	Benthic, locally abundant in shady habitats, 3-90 m deep.	Benthic, 7-60 m deep.
References	[14-19]	[18-21]	[22-24]	[25,26]	[27-29]

[a] This feature has also been described in the Pycnococcaceae [30].
[b] A decapore structure has also been described in *Pycnococcus provasolii*, although not as well developed [30]
[c] Studied using HPLC chromatography: see Latasa et al. [18] and Jouenne et al. [21] for a complete list of pigments.
[d] Studied using thin layer chromatography

Table S2. Taxon sampling for the plastid phylogeny.

Species	GenBank/EMBL Accession number(s)	Reference	Clade
Acetabularia acetabulum	HG518425-HG518474, HG794360	[31]	Chlorophyta
Acutodesmus obliquus	NC_008101	[32]	Chlorophyta
Bryopsis hypnoides	NC_013359	[33]	Chlorophyta
Chlamydomonas reinhardtii	NC_005353	[34]	Chlorophyta
Chlorella vulgaris	NC_001865	[35]	Chlorophyta
Coccomyxa sp.	NC_015084	[36]	Chlorophyta
Dunaliella salina	NC_016732	[37]	Chlorophyta
Floydiella terrestris	NC_014346	[38]	Chlorophyta
Leptosira terrestris	NC_009681	[39]	Chlorophyta
Micromonas sp. RCC 299	NC_012643	[40]	Chlorophyta
Monomastix sp. OKE-1	NC_012101	[41]	Chlorophyta
Nephroselmis astigmatica	KJ746600	[42]	Chlorophyta
Nephroselmis olivacea	NC_000927	[43]	Chlorophyta
Oedogonium cardiacum	NC_011031	[44]	Chlorophyta
Oltmannsiellopsis viridis	NC_008099	[45]	Chlorophyta
Oocystis solitaria	FJ968739	[46]	Chlorophyta
Ostreococcus tauri	NC_008289	[47]	Chlorophyta
Parachlorella kessleri	NC_012978	[46]	Chlorophyta
Pedinomonas minor	NC_016733	[46]	Chlorophyta
Picocystis salinarum	KJ746599	[42]	Chlorophyta
Prasinococcus capsulatus	AB491660, AB561011, AB561019, AB561027, AB561035, AB561044, AB561052, AB561060, AB561068, AB561076, AB561084	[48]	Chlorophyta
Prasinococcus sp. CCMP 1194	KJ746597	[42]	Chlorophyta
Prasinoderma coloniale	KJ746598	[42]	Chlorophyta
Prasinophyceae sp. CCMP 1205	KJ746601	[42]	Chlorophyta
Prasinophyceae sp. MBIC 10622	KJ746602	[42]	Chlorophyta
Pseudendoclonium akinetum	NC_008114	[49]	Chlorophyta
Pycnococcus provasolii	NC_012097	[41]	Chlorophyta
Pyramimonas parkeae	NC_012099	[41]	Chlorophyta
Schizomeris leibleinii	NC_015645	[50]	Chlorophyta
Stigeoclonium helveticum	NC_008372	[51]	Chlorophyta
Trebouxia aggregata	EU123962-EU124002	[52]	Chlorophyta
Verdigellas peltata	LT174527	This study	Chlorophyta
Volvox carteri	EU755264-EU755299	[53]	Chlorophyta
Adiantum capillus-veneris	NC_004766	[54]	Streptophyta
Anthoceros formosae	NC_004543	[55]	Streptophyta
Arabidopsis thaliana	NC_000932	[56]	Streptophyta
Chaetosphaeridium globosum	NC_004115	[57]	Streptophyta
Chara vulgaris	NC_008097	[58]	Streptophyta
Chlorokybus atmophyticus	NC_008822	[59]	Streptophyta

Cycas taitungensis	NC_009618	[60]	Streptophyta
Equisetum arvense	NC_014699	[61]	Streptophyta
Isoetes flaccida	NC_014675	[61]	Streptophyta
Klebsormidium flaccidum	KJ461680	[62]	Streptophyta
Mesostigma viride	NC_002186	[63]	Streptophyta
Mesotaenium endlicherianum	KJ461682	[62]	Streptophyta
Oryza sativa	NC_008155	[64]	Streptophyta
Physcomitrella patens	NC_005087	[65]	Streptophyta
Ptilidium pulcherrimum	NC_015402	[66]	Streptophyta
Roya anglica	KJ461681	[62]	Streptophyta
Staurastrum punctulatum	NC_008116	[67]	Streptophyta
Zygnema circumcarinatum	NC_008117	[67]	Streptophyta
Calliarthron tuberculosum	NC_021075	[68]	Rhodophyta
Chondrus crispus	NC_020795	[69]	Rhodophyta
Cyanidioschyzon merolae	NC_004799	[70]	Rhodophyta
Cyanidium caldarium	NC_001840	[71]	Rhodophyta
Gracilaria tenuistipitata	NC_006137	[72]	Rhodophyta
Grateloupia taiwanensis	NC_021618	[73]	Rhodophyta
Pyropia yezoensis	KC517072	[74]	Rhodophyta
Cyanophora paradoxa	NC_001675	[75]	Glaucophyta

Table S3. Taxon sampling for the nuclear rDNA phylogeny.

**Species**	**18S rDNA accession number**	**28S rDNA accession number**	**Clade**
Acrosiphonia arcta	AY303600	HQ603261	Chlorophyta
Chlorella vulgaris	AB162910	AB237642	Chlorophyta
Chlorochytridion tuberculatum	HE610134	HE610134	Chlorophyta
Crustomastix stigmatica	AJ629844	HE610148	Chlorophyta
Cymbomonas tetramitiformis	FN562438	HE610151	Chlorophyta
Desmochloris halophila	AB049416	HE610118	Chlorophyta
Dolichomastix tenuilepis	FN562449	HE610149	Chlorophyta
Dunaliella salina	DQ447646	DQ015739	Chlorophyta
Ignatius tetrasporus	FN562432	HE610121	Chlorophyta
Koliella longiseta	HE610126	HE610126	Chlorophyta
Leptosira terrestris	Z28973	Z95378	Chlorophyta
Mantoniella squamata	X73999	HE610150	Chlorophyta
Marsupiomonas pelliculata	HE610136	HE610137	Chlorophyta
Monomastix sp. M0722	FN562447	HE610147	Chlorophyta
Nephroselmis astigmatica	FN562433	HE610145	Chlorophyta
Nephroselmis olivacea	FN562436	HE610146	Chlorophyta
Nephroselmis pyriformis	X75565	HE610144	Chlorophyta
Oedogonium cardiacum	U83133	AF183478	Chlorophyta

Oltmannsiellopsis sp.	HE610120	HE610120	Chlorophyta
Ostreococcus tauri	CAID01000012	CAID01000012	Chlorophyta
Palmophyllum umbracola	FJ619275	-	Chlorophyta
Pedinomonas minor	HE610132	HE610132	Chlorophyta
Picocystis salinarum	AF125167	HE610138	Chlorophyta
Prasinococcus capsulatus	AF203401	HE610141	Chlorophyta
Prasinoderma coloniale	FN562437	HE610143	Chlorophyta
prasinophyte CCMP1205	U40921	HE610139	Chlorophyta
Pycnococcus provasolii	X91264	HE610140	Chlorophyta
Pyramimonas olivacea	FN562442	HE610153	Chlorophyta
Pyramimonas tetrarhynchus	FN562441	HE610152	Chlorophyta
Scenedesmus obliquus	AJ249513	AF183482	Chlorophyta
Tetraselmis striata	X70802	HE610129	Chlorophyta
Trebouxia usneae	Z68702	Z95385	Chlorophyta
Uronema belkae	AF182821	AF183489	Chlorophyta
Verdigellas peltata	LT174528	This study	Chlorophyta
Arabidopsis thaliana	CP002686	AC016828	Streptophyta
Chara globularis	Y16465	AJ271115	Streptophyta
Chlorokybus atmophyticus	M95612	AB491667	Streptophyta
Closterium ehrenbergii	AF115437	AF419992	Streptophyta
Coleochaete scutata	X68825	AB491669	Streptophyta
Mesostigma viride	AF408245	DQ980472	Streptophyta
Oryza sativa	CP012609	AA02002154	Streptophyta
Physcomitrella patens	AF223015	AC158196	Streptophyta
Cyanidioschyzon merolae	AB158483	AB158483	Rhodophyta
Pyropia yezoensis	KJ578747	KJ578748	Rhodophyta
Cyanophora paradoxa	AY823716	AY216932	Glaucophyta

References

1 R_Core_Team. R: A language and environment for statistical computing. R Foundation for Statistical Computing, Vienna, Austria. URL http://www.R-project.org/. (2016).

2 Pante, E. & Simon-Bouhet, B. marmap: A Package for Importing, Plotting and Analyzing Bathymetric and Topographic Data in R. *PLoS One* **8**, e73051 (2013).

3 Drummond, A. J. & Rambaut, A. BEAST: Bayesian evolutionary analysis by sampling trees. *BMC Evol. Biol.* **7**, 214 (2007).

4 Drummond, A. J., Suchard, M. A., Xie, D. & Rambaut, A. Bayesian phylogenetics with BEAUti and the BEAST 1.7. *Mol. Biol. Evol.* **29**, 1969-1973 (2012).

5 Yoon, H. S., Hackett, J. D., Ciniglia, C., Pinto, G. & Bhattacharya, D. A molecular timeline for the origin of photosynthetic eukaryotes. *Mol. Biol. Evol.* **21**, 809-818 (2004).

6 Hedges, S. B., Blair, J. E., Venturi, M. L. & Shoe, J. L. A molecular timescale of eukaryote evolution and the rise of complex multicellular life. *BMC Evol. Biol.* **4**, 2 (2004).

7 Douzery, E. J. P., Snell, E. A., Bapteste, E., Delsuc, F. & Philippe, H. The timing of eukaryotic evolution: Does a relaxed molecular clock reconcile proteins and fossils? *Proc. Natl Acad. Sci. U.S.A.* **101**, 15386-15391 (2004).

8 Berney, C. & Pawlowski, J. A molecular time-scale for eukaryote evolution recalibrated with the continuous microfossil record. *Proc. R. Soc. B* **273**, 1867-1872 (2006).
9 Roger, A. J. & Hug, L. A. The origin and diversification of eukaryotes: problems with molecular phylogenetics and molecular clock estimation. *Phil. Trans. R. Soc. B* **361**, 1039-1054 (2006).
10 Herron, M. D., Hackett, J. D., Aylward, F. O. & Michod, R. E. Triassic origin and early radiation of multicellular volvocine algae. *Proc. Natl Acad. Sci. U.S.A.* **106**, 3254-3258 (2009).
11 Parfrey, L. W., Lahr, D. J. G., Knoll, A. H. & Katz, L. A. Estimating the timing of early eukaryotic diversification with multigene molecular clocks. *Proc. Natl Acad. Sci. U.S.A.* **108**, 13624-13629 (2011).
12 Kenrick, P. & Crane, P. R. The origin and early evolution of plants on land. *Nature* **389**, 33-39 (1997).
13 Guiry, M. D. & Guiry, G. M. *AlgaeBase. World-wide electronic publication, National University of Ireland, Galway. http://www.algaebase.org; searched on 5 March 2016.*, <http://www.algaebase.org> (2016).
14 Miyashita, H., Ikemoto, H., Kurano, N., Miyachi, S. & Chihara, M. *Prasinococcus capsulatus* gen. et sp. nov., a new marine coccoid prasinophyte. *J. Gen. Appl. Microbiol.* **39**, 571-582 (1993).
15 Sieburth, J. M., Keller, M. D., Johnson, P. W. & Myklestad, S. M. Widespread occurrence of the oceanic ultraplankter, *Prasinococcus capsulatus* (Prasinophyceae), the diagnostic "Golgi-decapore complex" and the newly described polysaccharide "capsulan". *J. Phycol.* **35**, 1032-1043 (1999).
16 Guillou, L. *et al.* Diversity of picoplanktonic prasinophytes assessed by direct nuclear SSU rDNA sequencing of environmental samples and novel isolates retrieved from oceanic and coastal marine ecosystems. *Protist* **155**, 193-214 (2004).
17 Not, F. *et al.* A single species, *Micromonas pusilla* (Prasinophyceae), dominates the eukaryotic picoplankton in the western English channel. *Appl. Environ. Microbiol.* **70**, 4064-4072 (2004).
18 Latasa, M., Scharek, R., Le Gall, F. & Guillou, L. Pigment suites and taxonomic groups in Prasinophyceae. *J. Phycol.* **40**, 1149-1155 (2004).
19 Vaulot, D., Eikrem, W., Viprey, M. & Moreau, H. The diversity of small eukaryotic phytoplankton ($\leq$ 3 µm) in marine ecosystems. *FEMS Microbiol. Rev.* **32**, 795-820 (2008).
20 Hasegawa, T. *et al. Prasinoderma coloniale* gen. et sp. nov., a new pelagic coccoid prasinophyte from the western Pacific ocean. *Phycologia* **35**, 170-176 (1996).
21 Jouenne, F. *et al. Prasinoderma singularis* sp. nov. (Prasinophyceae, Chlorophyta), a solitary coccoid prasinophyte from the South-East Pacific Ocean. *Protist* **162**, 70-84 (2011).
22 Ballantine, D. L. & Norris, J. N. *Verdigellas*, a new deep-water genus (Tetrasporales, Chlorophyta) from the tropical western Atlantic. *Crypt. Bot.* **4**, 368-372 (1994).
23 Ballantine, D. L. & Aponte, N. E. *Verdigellas nektongammea* (Tetrasporales, Chlorophyta), a new deep-water species from the Bahamas. *Nova Hedwigia* **62**, 425-429 (1996).
24 Pueschel, C., Sullivan, K. & Ballantine, D. Ultrastructure of *Verdigellas peltata* (Palmellaceae, Chlorophyta), a deep-water, palmelloid alga with ferritin and trilaminar sheaths. *Phycologia* **36**, 492-499 (1997).
25 Nelson, W. A. & Ryan, K. G. *Palmophyllum umbracola* sp. nov. (Chlorophyta) from offshore islands of northern New Zealand. *Phycologia* **25**, 168-177 (1986).
26 Sartoni, G., Cinelli, F., Hirata, T., Katayama, N. & Yokohama, Y. On the lack of green light-harvesting pigments and the extremely high chlorophyll b/a ratio in the deep-water green alga, *Palmophyllum crassum* (Chlorosphaerales). *Jpn. J. Phycol.* **41**, 327-331 (1993).
27 Womersley, H. *Palmoclathrus*, a new deep water genus of Chlorophyta. *Phycologia* **10**, 229-233 (1971).
28 Womersley, H. B. S. *The Marine Benthic Flora of Southern Australia. Part I.* (Government Printer, South Australia, 1984).
29 O'Kelly, C. J. Division of *Palmoclathrus stipitatus* (Chlorophyta) vegetative cells. *Phycologia* **27**, 248-253 (1988).

30 Guillard, R. R., Keller, M. D., O'Kelly, C. J. & Floyd, G. L. *Pycnococcus provasolii* gen. et sp. nov., a coccoid prasinoxanthin-containing phytoplankter from the western north Atlantic and Gulf of Mexico. *J. Phycol.* **27**, 39-47 (1991).
31 de Vries, J. *et al.* Is ftsH the Key to Plastid Longevity in Sacoglossan Slugs? *Genome Biol. Evol.* **5**, 2540-2548 (2013).
32 de Cambiaire, J. C., Otis, C., Lemieux, C. & Turmel, M. The complete chloroplast genome sequence of the chlorophycean green alga *Scenedesmus obliquus* reveals a compact gene organization and a biased distribution of genes on the two DNA strands. *BMC Evol. Biol.* **6**, 37 (2006).
33 Lü, F. *et al.* The *Bryopsis hypnoides* plastid genome: Multimeric forms and complete nucleotide sequence. *PLoS One* **6**, e14663 (2011).
34 Maul, J. E. *et al.* The *Chlamydomonas reinhardtti* plastid chromosome: Islands of genes in a sea of repeats. *Plant Cell* **14**, 2659-2679 (2002).
35 Wakasugi, T. *et al.* Complete nucleotide sequence of the chloroplast genome from the green alga *Chlorella vulgaris*: The existence of genes possibly involved in chloroplast division. *Proc. Natl Acad. Sci. U.S.A.* **94**, 5967-5972 (1997).
36 Smith, D. R. *et al.* the GC-rich mitochondrial and plastid genomes of the green alga *Coccomyxa* give insight into the evolution of organelle DNA nucleotide landscape. *PLoS One* **6**, e23624 (2011).
37 Smith, D. R. *et al.* The *Dunaliella salina* organelle genomes: large sequences, inflated with intronic and intergenic DNA. *BMC Plant Biol.* **10**, 83 (2010).
38 Brouard, J.-S., Otis, C., Lemieux, C. & Turmel, M. The exceptionally large chloroplast genome of the green alga *Floydiella terrestris* illuminates the evolutionary history of the Chlorophyceae. *Genome Biol. Evol.* **2**, 240-256 (2010).
39 de Cambiaire, J. C., Otis, C., Turmel, M. & Lemieux, C. The chloroplast genome sequence of the green alga *Leptosira terrestris*: multiple losses of the inverted repeat and extensive genome rearrangements within the Trebouxiophyceae. *BMC Genomics* **8**, 213 (2007).
40 Worden, A. Z. *et al.* Green evolution and dynamic adaptations revealed by genomes of the marine picoeukaryotes *Micromonas*. *Science* **324**, 268-272 (2009).
41 Turmel, M., Gagnon, M.-C., O'Kelly, C. J., Otis, C. & Lemieux, C. The chloroplast genomes of the green algae *Pyramimonas*, *Monomastix*, and *Pycnococcus* shed new light on the evolutionary history of prasinophytes and the origin of the secondary chloroplasts of euglenids. *Mol. Biol. Evol.* **26**, 631-648 (2009).
42 Lemieux, C., Otis, C. & Turmel, M. Six newly sequenced chloroplast genomes from prasinophyte green algae provide insights into the relationships among prasinophyte lineages and the diversity of streamlined genome architecture in picoplanktonic species. *BMC Genomics* **15**, 857 (2014).
43 Turmel, M., Otis, C., Lemieux & C. The complete chloroplast DNA sequence of the green alga *Nephroselmis olivacea*: Insights into the architecture of ancestral chloroplast genomes. *Proc. Natl Acad. Sci. U.S.A.* **96**, 10248-10253 (1999).
44 Brouard, J.-S., Otis, C., Lemieux, C. & Turmel, M. Chloroplast DNA sequence of the green alga *Oedogonium cardiacum* (Chlorophyceae): Unique genome architecture, derived characters shared with the Chaetophorales and novel genes acquired through horizontal transfer. *BMC Genomics* **9**, 290 (2008).
45 Pombert, J. F., Lemieux, C. & Turmel, M. The complete chloroplast DNA sequence of the green alga *Oltmannsiellopsis viridis* reveals a distinctive quadripartite architecture in the chloroplast genome of early diverging ulvophytes. *BMC Biology* **4**, 3 (2006).
46 Turmel, M., Otis, C. & Lemieux, C. The chloroplast genomes of the green algae *Pedinomonas minor, Parachlorella kessleri*, and *Oocystis solitatia* reveal a shared ancestry between the Pedinomonadales and Chlorellales. *Mol. Biol. Evol.* **26**, 2317-2331 (2009).
47 Robbens, S. *et al.* The complete chloroplast and mitochondrial DNA sequence of *Ostreococcus tauri*: organelle genomes of the smallest eukaryote are examples of compaction. *Mol. Biol. Evol.* **24**, 956-968 (2007).
48 Matsumoto, T. *et al.* Green-colored plastids in the dinoflagellate genus *Lepidodinium* are of core chlorophyte origin. *Protist* **162**, 268-276 (2011).

49 Pombert, J. F., Otis, C., Lemieux, C. & Turmel, M. The chloroplast genome sequence of the green alga *Pseudendoclonium akinetum* (Ulvophyceae) reveals unusual structural features and new insights into the branching order of chlorophyte lineages. *Mol. Biol. Evol.* **22**, 1903-1918 (2005).

50 Brouard, J.-S., Otis, C., Lemieux, C. & Turmel, M. The chloroplast genome of the green alga *Schizomeris leibleinii* (Chlorophyceae) provides evidence for bidirectional DNA replication from a single origin in the Chaetophorales. *Genome Biol. Evol.* **3**, 505-515 (2011).

51 Bélanger, A. S. *et al.* Distinctive architecture of the chloroplast genome in the chlorophycean green alga *Stigeoclonium helveticum*. *Mol. Genet. Genomics* **276**, 464-477 (2006).

52 Pombert, J.-F. *et al.* Divergence order of chlorophyte green algal lineages as inferred from the chloroplast and mitochondrial genomes. *GenBank, unpublished* (2007).

53 Smith, D. R. & Lee, R. W. The mitochondrial and plastid genomes of *Volvox carteri*: bloated molecules rich in repetitive DNA. *BMC Genomics* **10**, 132 (2009).

54 Wolf, P. G., Rowe, C. A., Sinclair, R. B. & Hasebe, M. Complete nucleotide sequence of the chloroplast genome from a leptosporangiate fern, *Adiantum capillus-veneris* L. *DNA Res.* **10**, 59-65 (2003).

55 Kugita, M. *et al.* The complete nucleotide sequence of the hornwort (*Anthoceros formosae*) chloroplast genome: insight into the earliest land plants. *Nucleic Acids Res.* **31**, 716-721 (2003).

56 Sato, S., Nakamura, Y., Kaneko, T., Asamizu, E. & Tabata, S. Complete structure of the chloroplast genome of *Arabidopsis thaliana*. *DNA Res.* **6**, 283-290 (1999).

57 Turmel, M., Otis, C. & Lemieux, C. The chloroplast and mitochondrial genome sequences of the charophyte *Chaetosphaeridium globosum*: Insights into the timing of the events that restructured organelle DNAs within the green algal lineage that led to land plants. *Proc. Natl Acad. Sci. U.S.A.* **99**, 11275-11280 (2002).

58 Turmel, M., Otis, C. & Lemieux, C. The chloroplast genome sequence of *Chara vulgaris* sheds new light into the closest green algal relatives of land plants. *Mol. Biol. Evol.* **23**, 1324-1338 (2006).

59 Lemieux, C., Otis, C. & Turmel, M. A clade uniting the green algae *Mesostigma viride* and *Chlorokybus atmophyticus* represents the deepest branch of the Streptophyta in chloroplast genome-based phylogenies. *BMC Biology* **5**, 2 (2007).

60 Wu, C.-S., Wang, Y.-N., Liu, S.-M. & Chaw, S.-M. Chloroplast genome (cpDNA) of *Cycas taitungensis* and 56 cp protein-coding genes of *Gnetum parvifolium*: insights into cpDNA evolution and phylogeny of extant seed plants. *Mol. Biol. Evol.* **24**, 1366-1379 (2007).

61 Karol, K. G. *et al.* Complete plastome sequences of *Equisetum arvense* and *Isoetes flaccida*: implications for phylogeny and plastid genome evolution of early land plant lineages. *BMC Evol. Biol.* **10**, 321 (2010).

62 Civáň, P., Foster, P. G., Embley, T. M., Séneca, A. & Cox, C. J. Analyses of charophyte chloroplast genomes help characterize the ancestral chloroplast genome of land plants. *Genome Biol. Evol.* **6**, 897-911 (2014).

63 Lemieux, C., Otis, C. & Turmel, M. Ancestral chloroplast genome in *Mesostigma viride* reveals an early branch of green plant evolution. *Nature* **403**, 649-652 (2000).

64 Tang, J. *et al.* A comparison of rice chloroplast genomes. *Plant Physiol.* **135**, 412-420 (2004).

65 Sugiura, C., Kobayashi, Y., Aoki, S., Sugita, C. & Sugita, M. Complete chloroplast DNA sequence of the moss *Physcomitrella patens*: evidence for the loss and relocation of rpoA from the chloroplast to the nucleus. *Nucleic Acids Res.* **31**, 5324-5331 (2003).

66 Forrest, L. L., Wickett, N. J., Cox, C. J. & Goffinet, B. Deep sequencing of *Ptilidium* (Ptilidiaceae) suggests evolutionary stasis in liverwort plastid genome structure. *Plant. Ecol. Evol.* **144**, 29-43 (2011).

67 Turmel, M., Otis, C. & Lemieux, C. The complete chloroplast DNA sequences of the charophycean green algae *Staurastrum* and *Zygnema* reveal that the chloroplast genome underwent extensive changes during the evolution of the Zygnematales. *BMC Biology* **3**, 22 (2005).

68 Janouškovec, J. *et al.* Evolution of red algal plastid genomes: Ancient architectures, introns, horizontal gene transfer, and taxonomic utility of plastid markers. *PLoS One* **8**, e59001 (2013).

69 Collén, J. *et al.* Genome structure and metabolic features in the red seaweed *Chondrus crispus* shed light on evolution of the Archaeplastida. *Proceedings of the National Academy of Sciences* **110**, 5247-5252 (2013).
70 Ohta, N. *et al.* Complete sequence and analysis of the plastid genome of the unicellular red alga *Cyanidioschyzon merolae*. *DNA Res.* **10**, 67-77 (2003).
71 Glöckner, G., Rosenthal, A. & Valentin, K. The structure and gene repertoire of an ancient red algal plastid genome. *J. Mol. Evol.* **51**, 382-390 (2000).
72 Hagopian, J. C., Reis, M., Kitajima, J. P., Bhattacharya, D. & de Oliveira, M. C. Comparative analysis of the complete plastid genome sequence of the red alga *Gracilaria tenuistipitata* var. *liui* provides insights into the evolution of rhodoplasts and their relationship to other plastids. *J. Mol. Evol.* **59**, 464-477 (2004).
73 DePriest, M. S., Bhattacharya, D. & López-Bautista, J. M. The plastid genome of the red macroalga *Grateloupia taiwanensis* (Halymeniaceae). *PLoS One* **8**, E68246 (2013).
74 Wang, L. *et al.* Complete sequence and analysis of plastid genomes of two economically important red algae: *Pyropia haitanensis* and *Pyropia yezoensis*. *PLoS One* **8**, e65902 (2013).
75 Stirewalt, V. L., Michalowski, C. B., Löffelhardt, W., Bohnert, H. J. & Bryant, D. A. Nucleotide sequence of the cyanelle genome from *Cyanophora paradoxa*. *Plant Mol. Biol. Report.* **13**, 327-332 (1995).
